# Interplay between Notch1 and Notch3 promotes EMT and tumor initiation in squamous cell carcinoma

**DOI:** 10.1038/s41467-017-01500-9

**Published:** 2017-11-24

**Authors:** Mitsuteru Natsuizaka, Kelly A. Whelan, Shingo Kagawa, Koji Tanaka, Veronique Giroux, Prasanna M. Chandramouleeswaran, Apple Long, Varun Sahu, Douglas S. Darling, Jianwen Que, Yizeng Yang, Jonathan P. Katz, E. Paul Wileyto, Devraj Basu, Yoshiaki Kita, Shoji Natsugoe, Seiji Naganuma, Andres J. Klein-Szanto, J. Alan Diehl, Adam J. Bass, Kwok-Kin Wong, Anil K. Rustgi, Hiroshi Nakagawa

**Affiliations:** 1 Gastroenterology Division, Department of Medicine, University of Pennsylvania, Philadelphia, PA 19104 USA; 2 Abramson Cancer Center, Philadelphia, PA 19104 USA; 3 University of Pennsylvania Perelman School of Medicine, Philadelphia, PA 19104 USA; 4 Department of Gastroenterology and Hepatology, Hokkaido University Graduate School of Medicine, Sapporo, Hokkaido, 060-8638 Japan; 5 Department of General Surgery, Chiba University Graduate School of Medicine, Chiba, Chiba 260-0856 Japan; 6 Department of Surgery, Gastroenterological Surgery, Osaka University Graduate School of Medicine, Suita, Osaka, 565-0871 Japan; 7 Department of Otorhinolaryngology, University of Pennsylvania, Philadelphia, PA 19104 USA; 8 Department of Oral Immunology and Infectious Diseases, and Center for Genetics and Molecular Medicine, University of Louisville, Louisville, KY 40202 USA; 9 Department of Medicine, Division of Digestive and Liver Diseases, Columbia University, New York, NY 10032 USA; 10 Department of Biostatistics and Epidemiology, University of Pennsylvania, Philadelphia, PA 19104 USA; 11 Department of Digestive Surgery, Breast and Thyroid Surgery, Kagoshima University Graduate School of Medical and Dental Sciences, Kagoshima, 890-8520 Japan; 12 Department of Pathology, Kochi Medical School, Nankoku-shi, Kochi 783-8505 Japan; 13 Histopathology Facility and Cancer Biology Program, Fox Chase Cancer Center, Philadelphia, PA 19111 USA; 14 Department of Biochemistry and Molecular Biology, Hollings Cancer Center, Medical University of South Carolina, Charleston, SC 29425 USA; 15 Dana–Farber Cancer Institute, Department of Medicine, Harvard Medical School, Boston, MA 02215 USA; 16 Division of Hematology and Medical Oncology, New York University, New York, NY 10016 USA

## Abstract

Notch1 transactivates *Notch3* to drive terminal differentiation in stratified squamous epithelia. Notch1 and other Notch receptor paralogs cooperate to act as a tumor suppressor in squamous cell carcinomas (SCCs). However, Notch1 can be stochastically activated to promote carcinogenesis in murine models of SCC. Activated form of Notch1 promotes xenograft tumor growth when expressed ectopically. Here, we demonstrate that Notch1 activation and epithelial–mesenchymal transition (EMT) are coupled to promote SCC tumor initiation in concert with transforming growth factor (TGF)-β present in the tumor microenvironment. We find that TGFβ activates the transcription factor ZEB1 to repress *Notch3*, thereby limiting terminal differentiation. Concurrently, TGFβ drives Notch1-mediated EMT to generate tumor initiating cells characterized by high CD44 expression. Moreover, Notch1 is activated in a small subset of SCC cells at the invasive tumor front and predicts for poor prognosis of esophageal SCC, shedding light upon the tumor promoting oncogenic aspect of Notch1 in SCC.

## Abstract (web summary)

Notch receptors can exert different roles in cancer. In this manuscript, the authors reveal that Notch1 activation and EMT promote tumor initiation and cancer cell heterogeneity in squamous cell carcinoma, while the repression of Notch3 by ZEB1 limits Notch1-induced differentiation, permitting Notch1-mediated EMT.

## Introduction

Notch signaling regulates cell fate in a context-dependent manner^1^. The ligand-activated intracellular domain of Notch (ICN) forms a transcriptional activation complex with the transcription factor CSL and the co-activator MAML. Notch1 drives terminal differentiation in stratified squamous epithelia^2, 3^ in concert with other Notch receptor paralogs^4, 5^. Histopathology of squamous cell carcinomas (SCCs) features squamous-cell differentiation, a process normally regulated via direct transcriptional activation of *Notch3* by ICN1, the activated form of Notch1, in esophageal epithelia^4^. Loss-of-function Notch1 mutations are found in SCCs^6, 7^, suggesting a tumor suppressor role for Notch1^5, 8, 9^. However, Notch1 can be stochastically activated or inactivated, with either scenario resulting in promotion of carcinogenesis in murine models of SCC^10^. Many human SCC cell lines express ICN1 and ectopic ICN1 expression promotes xenograft tumor growth^11, 12^. While pharmacological modulation of Notch paralogs represents an attractive strategy for cancer therapy^13^, a more detailed understanding of the functional role of the Notch pathway as it relates to tissue biology in the context of health and disease is necessary to guide such approaches.

In addition to squamous-cell differentiation, Notch1 regulates cell cycle^3, 12, 14^, senescence^12^, and epithelial–mesenchymal transition (EMT)^15–17^. Acquisition of mesenchymal properties facilitates malignant transformation by limiting oncogene-induced senescence^18, 19^. In human esophageal squamous cell carcinoma (ESCC), the deadliest form of all human SCCs^20^, EMT is associated with chemoresistance and poor prognosis^21–23^. EMT also regulates cancer stem cells (CSCs)^24, 25^. CSCs defined by high CD44 expression (CD44H) have been identified in various tumor types, including SCCs^26–29^. In transformed esophageal and oral keratinocytes, cells with low CD44 expression (CD44L) and epithelial properties are converted to CD44H cells with mesenchymal traits in response to transforming growth factor (TGF)-β^30, 31^, a potent EMT inducer present in the tumor microenvironment^32^. During TGFβ-mediated EMT, expression of the Notch ligand JAG1 is induced via ZEB1^12, 15, 33^, a transcription factor essential in TGFβ-induced EMT^34, 35^ and microRNA-mediated regulation of Notch signaling^33, 36, 37^. While emerging lines of evidence support Notch1 as a positive effector of EMT^15–17, 37, 38^, Notch3 limits the expansion of EMT-competent esophageal keratinocytes^11^. Thus, although Notch1 and Notch3 cooperate to drive squamous-cell differentiation^4^, these Notch paralogs may play opposing roles in EMT and, potentially, regulation of CSC dynamics. The precise molecular mechanisms through which Notch signaling regulates distinct cell fates in a context-dependent manner have yet to fully elucidated.

Here, we aimed to define the functional role of Notch1 in SCC. We demonstrate that Notch1 activation and EMT are coupled to promote tumor initiation and intratumoral cancer cell heterogeneity in SCC. We find that the transcription factor ZEB1 represses *NOTCH3*, thereby limiting ICN1-induced differentiation while permitting ICN1-mediated EMT. Moreover, ICN1 expression in a small subset of SCC cells at the invasive tumor front predicts independently for poor prognosis of ESCC. These findings suggest an oncogenic role for Notch1 in SCC and identify the TGFβ–ZEB1–Notch1 axis as potential target for SCC therapy.

## Results

### EMT and Notch1 activation are features of carcinogen-driven ESCC in vivo

**Fig. 1:**
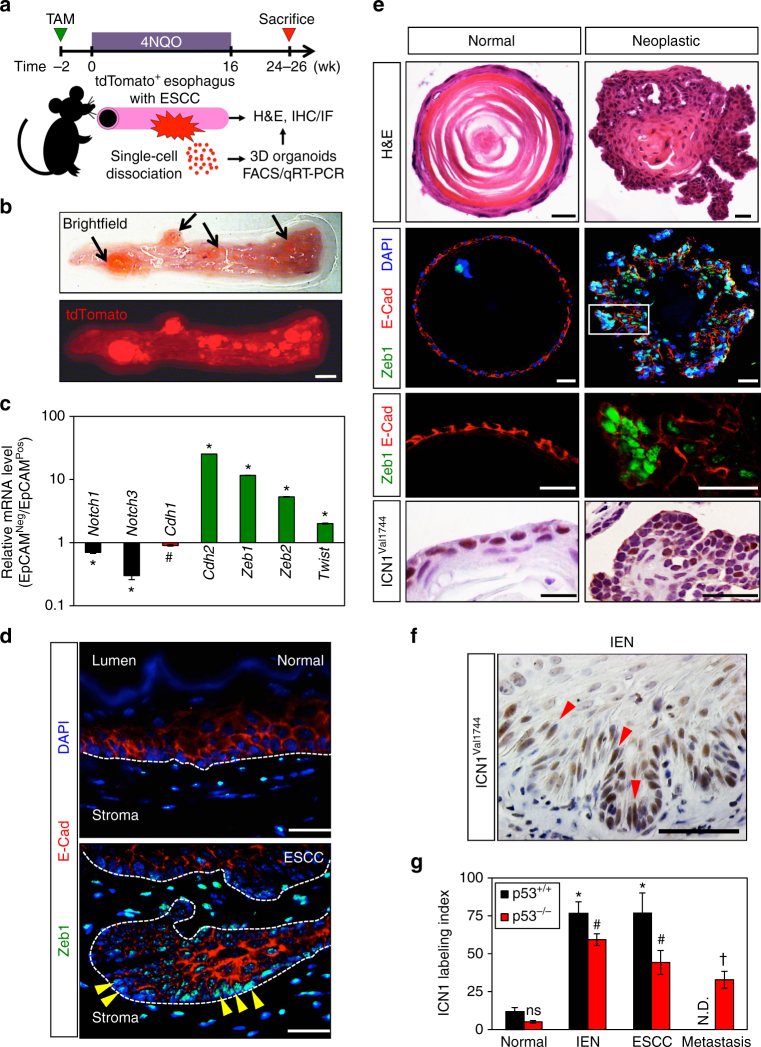
Lineage tracing reveals mesenchymal traits and Notch1 activation in ESCC lesions. **a** Schematic of cell-lineage tracing experiments. **b** Macroscopic and fluorescent images of representative tdTomato-labeled esophagus bearing 4NQO-induced tumors (arrows). Scale bar, 1 mm. **c** qRT-PCR analysis for indicated genes comparing EpCAM^neg^ to EpCAM^pos^ cells from a representative tdTomato-labeled tumor. *P < 0.0001 and ^#^ *P* < 0.05 vs. EpCAM^pos^, *n* = 3. **d** Representative IF images for Zeb1 and E-cadherin (E-cad) in normal mucosa (top) and ESCC (bottom). Dashed line denotes interface between stroma and basal keratinocytes (top) or invasive ESCC cells (bottom). Note E-cadherin downregulation in ESCC cells with nuclear Zeb1 (arrowheads) at the tumor invasive front. Scale bar, 50 µm. **e** H&E, multicolor IF for E-cadherin and Zeb1, and IHC for ICN1 (ICN1^Val1744^) in representative single-cell-derived organoids from normal mucosa or 4NQO-induced tumors. Note that spherical organoids from 4NQO-untreated control mice exhibit a differentiation gradient with predominant luminal keratinization, whereas tumor-derived organoids (neoplastic) display irregular morphology with increased cellularity and diminished keratinization. Zeb1 expression was robust in tumor organoids, particularly at invasive protrusions with decreased E-cadherin expression and detectable ICN1 expression. Box denotes area magnified in panel below. Scale bars, 20 µm. **f** Representative IHC image for ICN1 4NQO-induced IEN (dysplasia) containing spindle-shaped cells (arrowheads). Scale bar, 50 µm. **g** Quantification of ICN1 IHC scoring in normal mucosa and 4NQO-induced lesions in mice of indicated genotype. *P < 0.05 vs. *p53* ^+/+^ (*K5Cre* ^*ERT2*^ *;R26tdTomato* ^*lsl/lsl*^) normal, ^#^ *P* < 0.0005 vs. *p53* ^*−/−*^ (*K5Cre* ^*ERT2*^ *;R26tdTomato* ^*lsl/lsl*^; *p53* ^*loxP/loxP*^) normal; ^†^ *P* < 0.05 vs. *p53* ^*−/−*^ IEN; ns not significant vs. *p53* ^*+/+*^ normal. Data in **b**–**f** represent at least three independent 4NQO-induced lesions and >20 organoids from at least two independent experimental replicates. In **g**, *n* = 5 normal, *n* = 8 IEN, and *n* = 5 invasive ESCC in p53^+/+^ esophagi. N.D. not detectable. *n* = 3 normal, *n* = 7 IEN, *n* = 5 invasive ESCC, and *n* = 4 metastatic tumors in p53^−/−^ esophagi. Two independent experimental replicates were carried out. Bar diagrams represent mean ± s.d. in **c** and mean ± s.e.m. in **g**. Student’s *t*-test was used for paired data comparisons in **c**, **g**. ANOVA with Tukey’s post hoc test was used for multiple comparisons in **g**

To study SCC initiation and progression in vivo, we treated mice with 4-Nitroquinoline 1-oxide (4NQO), a potent oral-esophageal carcinogen. We combined 4NQO treatment with a cell-lineage tracing experimental system in which murine oral and esophageal epithelial basal cells (keratinocytes) were marked permanently with tdTomato fluorescent protein following tamoxifen (TAM)-induced Cre-mediated recombination in *K5Cre* ^*ERT2*^ *;R26tdTomato* ^*lsl/lsl*^ mice (Fig. 1a). 4NQO-induced lesions showed tdTomato accumulation (Fig. 1b; Supplementary Fig. 1a, b), validating the basal keratinocyte origin of these tumors^39^. Flow cytometry revealed the presence of cells displaying both negative and positive expression of EpCAM (EpCAM^neg^ and EpCAM^pos^), an epithelial cell surface marker, within the tdTomato-positive (tdTomato^pos^) fractions of 4NQO-induced ESCC lesions (Supplementary Fig. 1c), suggesting a loss of epithelial characteristics in tumor cells originating from esophageal basal keratinocytes. In cell lineage tracing experiments, tdTomato expression assures that these EpCAM^neg^ cells are not co-existing intratumoral stromal cells (e.g., fibroblasts) which are not labeled with tdTomato via *K5Cre* ^*ERT2*^. Quantitative reverse-transcription polymerase chain reaction (qRT-PCR) analysis coupled with fluorescence-activated cell sorting (FACS) demonstrated further upregulation of genes associated with mesenchymal cells (Fig. 1c) including *Zeb1*, in tdTomato^pos^;EpCAM^neg^ cells originating from basal esophageal keratinocytes. Consistent with EMT as described in human ESCC^11^, neoplastic lesions displayed E-cadherin loss or mislocalization as well as robust Zeb1 expression at the stromal interface (Fig. 1d). Single-cell-derived three-dimensional (3D) organoids generated from 4NQO-induced dysplastic mucosa and primary tumors recapitulated acquisition of mesenchymal properties as found in 4NQO-induced neoplastic lesions (Fig. 1e). We also included mice with conditional loss or expression of mutant p53 since p53 dysfunction promotes EMT^19, 31, 40^ and found that more pronounced E-cadherin loss and reciprocal Zeb1 upregulation were observed in invasive tumors with mutant p53^R172H^ (Supplementary Fig. 1d).

We next utilized this experimental platform to examine expression of ICN1 (ICN1^Val1744^), the activated form of Notch1, throughout the natural history of ESCC. In comparison to normal esophageal squamous epithelia, ICN1 expression was increased in dysplastic lesions and remained upregulated in primary and metastatic ESCC (Fig. 1f, g; Supplementary Fig. 1e). Moreover, ICN1 was detected in invasive ESCC cells displaying E-cadherin mislocalization and Zeb1 upregulation (Supplementary Fig. 1d). ICN1 was also expressed highly in neoplastic organoids displaying EMT (Fig. 1e), suggesting a potential role for ICN1 in the acquisition of mesenchymal properties by SCC cells. The tumor suppressor p53 protein can transcriptionally activate *Notch1* in response to genotoxic stress^41^. Since 4NQO activates *p53* via DNA damage^42^, ICN1 expression in 4NQO-induced early lesions may reflect p53 activation in dysplastic cells. Conversely, Notch1 downregulation may be accounted for by p53 inactivation during disease progression. Therefore, we evaluated the influence of p53 loss upon ICN1 expression in the esophageal epithelium. TAM-induced *K5Cre* ^*ERT2*^-driven *p53* loss did not affect ICN1 expression in mice without 4NQO treatment (*P* = 0.06 by Student’s *t*-test; *n* = 3 animals per group; two independent experimental replicates), a finding that recapitulates p53^−/−^ murine epidermis^43^. When *p53* was deleted in oral-esophageal keratinocytes then mice were treated with 4NQO, *p53* loss did not prevent ICN1 expression in neither normal esophageal epithelium nor 4NQO-induced neoplastic lesions (Fig. 1g; Supplementary Fig. 1e). Mice with *p53* deletion did, however, display frequent metastases (Supplementary Fig. 1a). These findings suggest that p53 may be dispensable for Notch1 activation in 4NQO-induced lesions, albeit essential for overall SCC progression.

### Notch1 promotes ESCC tumorigenicity and expansion of CD44H cells with mesenchymal properties

**Fig. 2:**
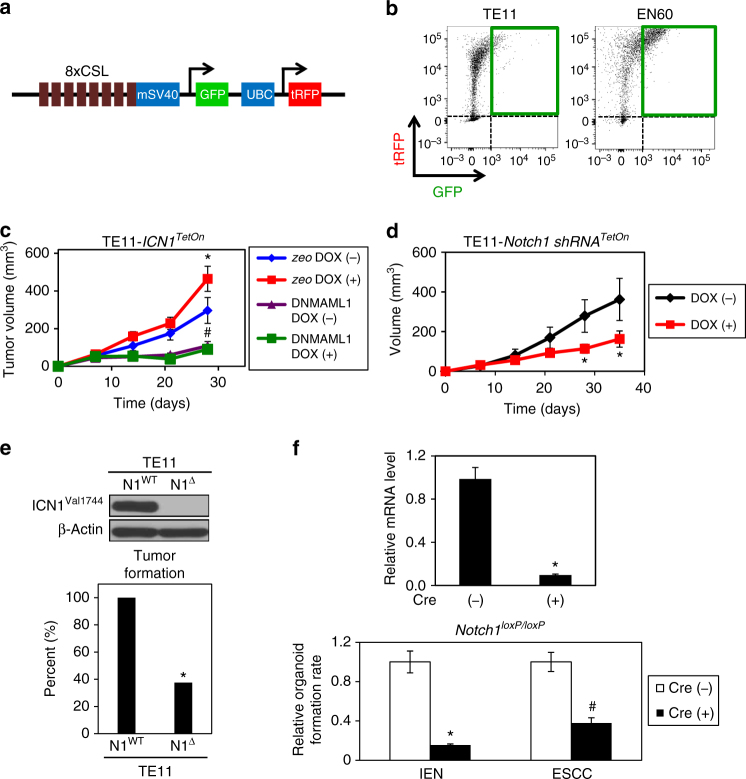
Notch1 promotes ESCC tumorigenesis. **a** Schematic of *8×CSL-GFP* reporter. Notch activation permits GFP reporter expression via concatemeric CSL-binding sites. Constitutively active UBC promoter drives tRFP expression concurrently. Cells without tRFP represent host-derived stromal cells. **b** Representative flow cytometry scatter plots determining *8×CSL*-mediated GFP reporter activation, indicating Notch active population of tRFP-labeled ESCC cells in TE11 and EN60 xenograft tumors. On average, 43.5 ± 0.5% s.d. of EN60 and 11.2 ± 6.5% s.d. of TE11 cells comprised the live GFP^Pos^/tRFP^Pos^ fraction across three independent tumors. **c**, **d** Tumor growth curves in immunodeficient mice carrying TE11 tumors of indicated genotypes. Upon xenograft transplantation, mice were treated with DOX to induce *ICN1* or *NOTCH*1-directed shRNA. Tumor growth was monitored for indicated time periods. In **c**, *P < 0.01 vs. *zeo* and DOX (−), ^#^ *P* < 0.05 vs. *zeo* and DOX (+), *n* = 6–8 per group. In **d**, *P < 0.05 vs. DOX (−); *n* = 6 per group. **e** TE11 cells with CRISPR/Cas9-mediated *NOTCH1* deletion. Immunoblotting confirms ICN1 loss in *NOTCH1*-deleted TE11 (N1^Δ^), but not non-targeted control TE11 (N1^WT^) cells. Bar graph shows tumor formation rate in immunodeficient mice 7 weeks after xenograft transplantation. *P < 0.05 vs. N1^WT^, *n* = 8 per group. **f** Esophageal keratinocytes isolated from 4NQO-induced IEN (dysplasia) lesions or ESCC tumors of *Notch1* ^*loxP/loxP*^ mice were utilized to generate 3D esophageal organoids in the presence or absence of ex vivo Cre-mediated recombination. qRT-PCR analysis confirms inhibition of *Notch1* gene expression upon ex vivo Cre-mediated recombination in ESCC organoids. *P < 0.000 vs. Cre (−), *n* = 3. Organoid formation rate was evaluated at day 14 post-plating and is represented as relative to Cre (−) for IEN and ESCC. *P < 0.000 vs. Cre (−) IEN; ^#^ *P* < 0.001 vs. Cre (−) ESCC. Line graphs represent mean ± s.e.m. in **c**, **d**. Bar diagrams represent mean ± s.d. in **f**. At least two independent replicates were performed for all experiments. Repeated measures ANOVA with Tukey’s post hoc test were used for multiple comparisons in **c**, **d**. Chi square with Fisher’s exact test was used for percentage comparisons in **e**. Student’s *t*-test was used for paired data comparisons in **f**

To explore further the functional role of Notch1 in ESCC tumorigenicity, we first utilized the extensively characterized human ESCC cell lines TE11 and EN60^12^. Both express ICN1 and form tumors upon xenograft transplantation in immunodeficient mice where doxycycline (DOX)-inducible ectopic ICN1 augments tumor growth^11, 12^. To assess Notch activity in vivo, we used *8×CSL-GFP*, a lentiviral green fluorescent protein (GFP) reporter driven by the minimal SV40 promoter fused to concatemeric CSL-binding sites (Fig. 2a). In TE11 and EN60 xenograft tumors, a subset of turboRFP (tRFP)-expressing tumor cells exhibited CSL-mediated transcriptional activity (Fig. 2b). A functional role for Notch activity in ESCC tumor growth is evident as dominant negative mutant *MAML1* (*DNMAML1*)^44^ prevented DOX-induced ectopic ICN1 from stimulating tumor growth (Fig. 2c). Additionally, DNMAML1 alone or *NOTCH1*-directed short hairpin RNA (shRNA) suppressed tumor volume significantly (Fig. 2c, d). CRISPR/Cas9-mediated *NOTCH1* deletion dramatically impaired tumor formation by TE11 cells in immunodeficient mice (Fig. 2e), supporting a role for Notch1 in tumor initiation as well. EN60 cells are less tumorigenic than TE11 upon xenograft transplantation, displaying 12.5–25% tumor formation rates with 1–4 × 10^6^ cells transplanted; however, DOX-induced ectopic ICN1 enhanced EN60 tumor formation rate to 100% (*P* < 0.01 by Fisher’s exact test; *n* = 12; two independent experimental replicates). Moreover, the ability of esophageal neoplastic cells to form single-cell-derived 3D organoids was attenuated upon Cre-mediated ex vivo Notch1 deletion in single-cell suspensions prepared from dysplastic lesions or ESCC tumors of 4NQO-treated *Notch1* ^*loxP/loxp*^ mice (Fig. 2f).

**Fig. 3:**
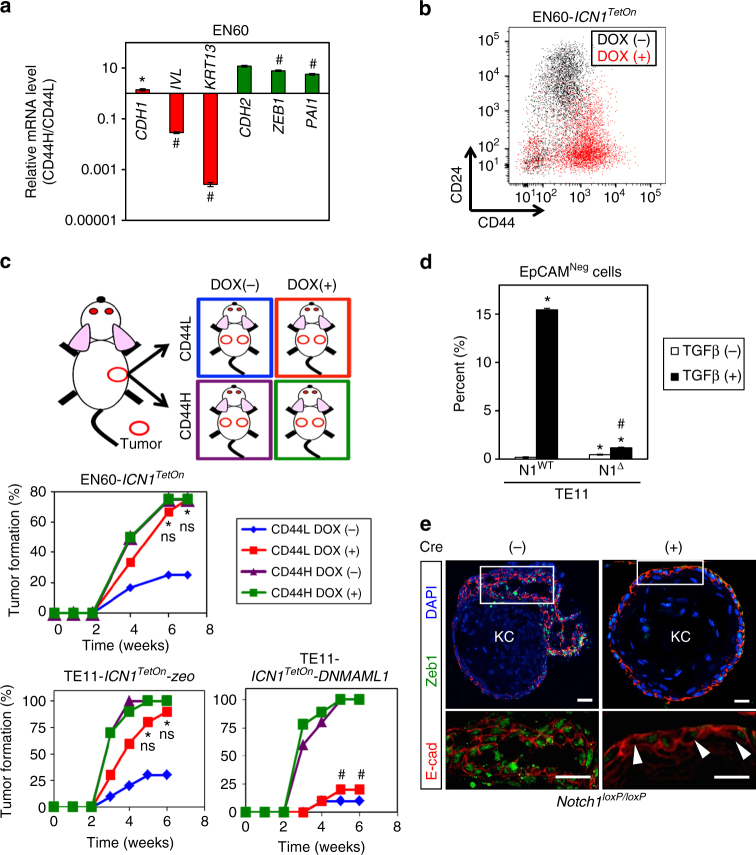
Notch1 facilitates expansion of CD44H cells with mesenchymal properties. **a** qRT-PCR analysis for indicated genes comparing EN60 intratumoral CD44L and CD44H cells. *P < 0.05 vs. CD44L; ^#^ *P* < 0.0001 vs. CD44L; *n* = 3 per group. **b** Representative flow cytometry scatter plot determining CD44H cells in EN60 tumors grown for 4 weeks with or without DOX-induced ICN1 (EN60-*ICN1* ^*TetOn*^). **c** Experimental design of serial transplantation experiments and tumor formation rates by CD44L and CD44H cells purified from parental xenograft tumors carrying DOX-inducible *ICN1* (EN60-*ICN1* ^*TetOn*^ and TE11-*ICN1* ^*TetOn*^). Parental tumors were grown in mice without DOX treatment and dissociated for FACS-purification of CD44L and CD44H cells. Purified cells were serially transplanted (10^3^ cells per injection site) into recipient mice to monitor tumor formation. Recipient mice were treated with or without DOX. TE11 carried *DNMAML1* or *zeo* (empty vector control). *P < 0.05 for CD44L and DOX (+) vs. CD44L and DOX (−), *n* = 10–12 per group; ns not significant for CD44L and DOX (+) vs. CD44H (with or without DOX treatment), *n* = 10 per group; ^#^ *P* < 0.05 vs. TE11-*ICN1* ^*TetOn*^ *-zeo* CD44L and DOX (+), *n* = 10 per group. **d** Flow cytometry analysis for EpCAM-negative cells in TE11 with *NOTCH1* deletion (N1^Δ^) or wild-type control (N1^WT^). Cells were treated with or without 5 ng/ml TGFβ for 72 h. *P < 0.05 vs. TGFβ (−); ^#^ *P* < 0.0001 vs. N1^WT^ and TGFβ (+), *n* = 3. Data are presented as mean ± s.e.m. **e** Multicolor IF for E-cadherin and Zeb1 in representative single-cell-derived organoids from 4NQO-induced ESCC tumors of *Notch1* ^*loxP/loxP*^ mice with or without ex vivo Cre-mediated recombination. Zeb1 expression diminished in organoids upon Cre-mediated Notch1 deletion (arrowheads). Box denotes area that is magnified in panel below. KC keratinized core of organoids. Scale bars, 20 µm. Bar diagrams represent mean ± s.d. in **a**, **d**. At least two independent replicates were performed for all experiments. Student’s *t*-test was used for paired data comparisons in **a**, **d**. Chi square with Fisher’s exact test was used for percentage comparisons in **c**

Human SCC tumors comprise CD44L and CD44H cells, the latter exhibiting augmented tumor-initiating capability^28, 29^. CD44H expression is associated with mesenchymal characteristics in transformed oral-esophageal cell lines^31^. Following FACS-purification, early passage CD44H TE11 and EN60 cells maintained enhanced expression of the mesenchymal cell marker N-cadherin as compared to their CD44L counterparts in culture (Supplementary Fig. 2a). Additionally, CD44H cells isolated from TE11 and EN60 xenograft tumors exhibited upregulation of *ZEB1* and *CDH2* (N-cadherin) (Fig. 3a; Supplementary Fig. 2b). As ectopic ICN1 expression in EN60 tumors enhanced robustly intratumoral CD44H cell content (Fig. 3b), we hypothesized that Notch1 contributes to tumor initiation via generation of CD44H cells that have mesenchymal properties. To determine how Notch1 may influence tumor initiation by CD44L and CD44H cells, we performed serial transplantation experiments (Fig. 3c). We first grew tumors without activating DOX-inducible ICN1. We then purified CD44L and CD44H cells by FACS from primary tumors and 1 × 10^3^ cells were serially injected into recipient mice where DOX was given to induce ectopic ICN1 expression. CD44L cells purified from EN60 and TE11 tumors showed low (<30%) spontaneous tumor formation efficiency; however, ICN1 dramatically stimulated tumor initiation by CD44L cells to 80–90%. In TE11 CD44L cells, DNMAML1 not only antagonized ICN1-mediated tumorigenicity, but also suppressed spontaneous tumor formation. While purified CD44H cells were highly tumorigenic (80–100%), neither ectopic ICN1 nor DNMAML1 affected tumor initiation by CD44H cells, suggesting that established CD44H-mediated tumor initiation is independent of Notch1. To our knowledge, this is the first demonstration that Notch1 may facilitate tumor initiation by converting CD44L cells to highly tumorigenic CD44H cells with mesenchymal traits in vivo.

### TGFβ and Notch1 cooperate to drive EMT in the tumor microenvironment

The functional role of Notch1 in EMT was suggested as *NOTCH1* deletion in cultured TE11 cells attenuated sharply TGFβ-mediated EpCAM^neg^ cell induction (Fig. 3d). Cre-mediated ex vivo *Notch1* deletion in 3D ESCC organoids generated from 4NQO-induced *Notch1* ^*loxP/loxP*^ murine tumors resulted in a diminished expression of Zeb1 (Fig. 3e). Moreover, DOX-induced ectopic ICN1 augmented intratumoral EpCAM^neg^ cell content in TE11 xenograft tumors (Supplementary Fig. 2c). While these data support a role for Notch1 in promotion of ESCC tumor cells with attributes of EMT, EpCAM^neg^ cells were rare in cultured TE11 cells and ectopic ICN1 expression alone had no influence upon this cell population (Supplementary Fig. 2c), indicating that Notch1 activation alone may not be sufficient to drive EMT.

**Fig. 4:**
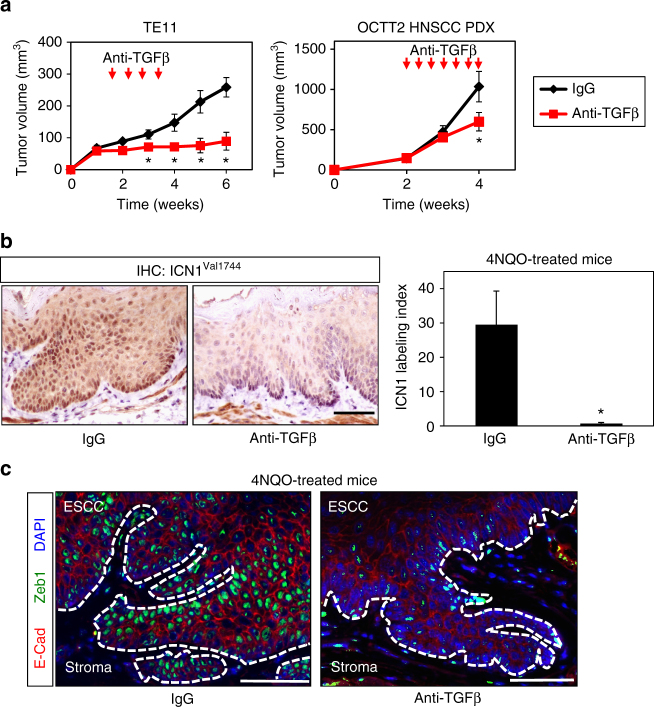
TGFβ signaling facilitates ESCC tumor growth, Notch1 signaling, and EMT. **a** Growth curve for mice bearing TE11 or HNSCC PDX tumors receiving either ID11 anti-TGFβ blocking antibody or control IgG via intraperitoneal injection at indicated time points (arrows). ID11 neutralizes all TGFβ isoforms. *P < 0.05 vs. IgG, *n* = 5–8 per group. **b**, **c** *p53* ^*+/+*^ mice were treated with 4NQO for 16 weeks. Six weeks following 4NQO withdrawal, mice were treated with ID11 anti-TGFβ blocking antibody or control IgG via intraperitoneal injection three times weekly for a period of 2 weeks. In **b**, quantification of ICN1 IHC scoring in esophageal epithelium with representative IHC images for IgG-treated and anti-TGFβ blocking antibody-treated animals. *P < 0.05; *n* = 4–5 per group. Scale bar, 50 µm. In **c**, multicolor IF for E-cadherin and Zeb1 in representative 4NQO-induced IEN lesions from IgG-treated and anti-TGFβ blocking antibody-treated animals. Data indicate mean ± s.e.m. in **a**, **b**. At least two independent replicates were performed for all experiments. Repeated measures ANOVA with Tukey’s post hoc test were used for multiple comparisons in **a**. Student’s *t*-test was used for paired data comparisons in **b**

In the tumor microenvironment, Notch signaling may be modulated by other transcription factors such as HIF1α^17^ and SMAD3^45^ via physical interactions with ICN1. We suspected that TGFβ influences Notch1-mediated tumor promotion and EMT since the TGFβ target gene *PAI1* was upregulated in CD44H cells in tumors (Fig. 3a; Supplementary Fig. 2b). In agreement, an anti-TGFβ blocking therapeutic monoclonal antibody 1D11^46^ severely impaired tumor growth of TE11 xenograft tumors as well as OCTT2 head and neck SCC patient-derived xenografts (PDXs) (Fig. 4a). In the context of 4NQO-mediated carcinogenesis, the percentage of esophageal mucosa occupied by neoplastic lesions classified as intraepithelial neoplasia (IEN) or ESCC was diminished upon treatment with 1D11 (26.0% ± 8.1 s.e.m.; *n* = 5) as compared to isotype control (56.3% ± 14.2 s.e.m.; *n* = 4; *P* = 0.09 by Student’s *t*-test; two independent experimental replicates). Administration of 1D11 to 4NQO-treated animals further attenuated ICN1 expression as well as morphological evidence of EMT in esophageal epithelium (Fig. 4b, c). Taken together, these data indicate that TGFβ functions as a critical positive effector of Notch1 and EMT in the context of the tumor microenvironment.

**Fig. 5:**
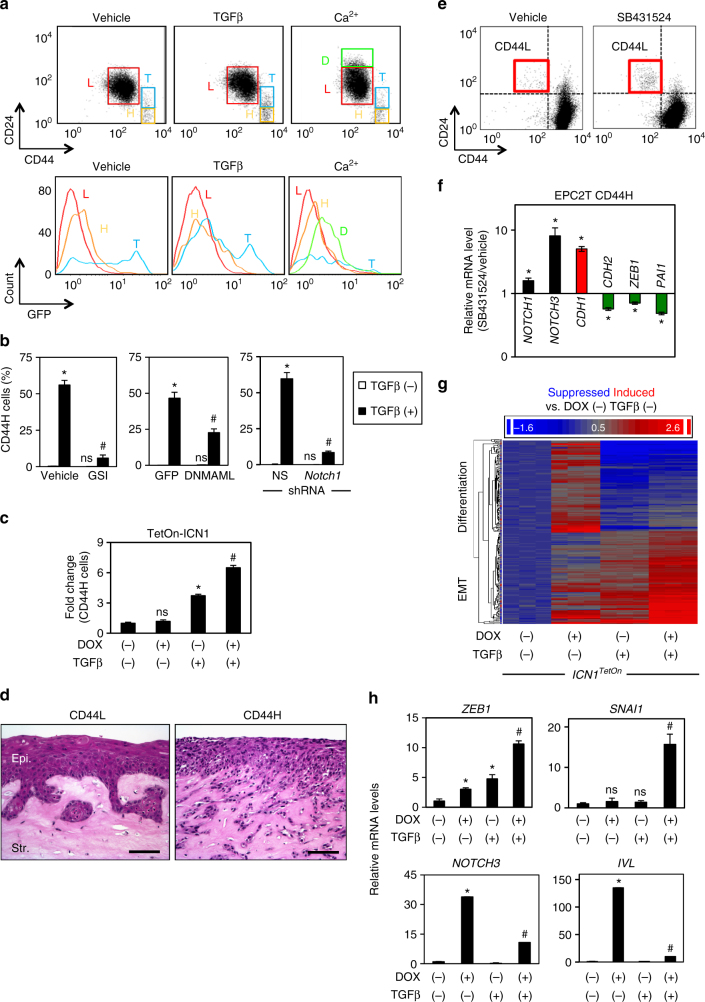
TGFβ-mediated Notch1 activation permits EMT and suppresses differentiation. **a** Representative flow cytometry scatter and histogram plots for *8×CSL-GFP* reporter activity in indicated subpopulations of EPC2T cells treated with TGFβ or CaCl_2_ for 72 h. **b**, **c** Flow cytometry determined CD44H cells induced in FACS-purified CD44L cells with indicated conditions or genotypes. Cells were treated with TGFβ or DOX for 7 days. *P < 0.0001 vs. TGFβ (−); ^#^ *P* < 0.005 vs. TGFβ (+) and DMSO, *GFP*, or non-silencing scrambled (NS) shRNA in **b**; *n* = 3. *P < 0.05 vs. TGFβ (−) and DOX (−); ns not significant vs. TGFβ (−) and DOX (−); ^#^ *P* < 0.05 vs. TGFβ (+) and DOX (−) in **c**; *n* = 3. **d** Representative H&E images of OTC reconstituted with CD44L or CD44H cells. Epi epithelia, Str stroma. Scale bar, 50 μm. **e** Representative flow cytometry scatter plots of purified CD44H cells treated with SB431542 or vehicle (control) for 14 days. SB431542 increased CD44L cell content (upper left quadrant) to 0.35 ± 0.08% as compared to 1.2 ± 0.1% s.d. in vehicle (*P* < 0.0005, *n* = 3). **f** qRT-PCR analysis for indicated genes in FACS-purified CD44H cells treated with or without SB431542. mRNA level for each gene in vehicle-treated cells was set as 1. *P < 0.0005 vs. vehicle, *n* = 3. **g** Heat map of gene array results from EPC2T derivative (EPC2T-*ICN1* ^*TetOn*^) treated with DOX and/or TGFβ (*n* = 3 per condition). “Differentiation” genes were significantly increased (≥1.5-fold) by DOX-induced ectopic ICN1 alone, but suppressed by TGFβ (*P* < 0.05 by two-way ANOVA). “EMT” genes activated by TGFβ (≥1.5-fold) were significantly augmented by ectopic ICN1 (*P* < 0.05 by two-way ANOVA). **h** qRT-PCR analysis validates gene array results in **g**. mRNA level for DOX (−) and TGFβ (−) was set to 1. *P < 0.001 vs. TGFβ (−) and DOX (−); ^#^ *P* < 0.0001 vs. TGFβ (−) and DOX (+); ns not significant vs. TGFβ (−) and DOX (−); *n* = 3. All bar diagrams indicate mean ± s.d. Student’s *t*-test was used for paired data comparisons in **e**, **f**. At least two independent replicates were performed for all experiments. ANOVA with Tukey’s post hoc test were used for multiple comparisons in **b**, **c**, **h**

To dissect further the mechanistic role of Notch1 in the generation and maintenance of ESCC tumor cells with mesenchymal features, we utilized the genetically engineered transformed esophageal cell line EPC2T (EPC2-hTERT-EGFR-p53^R175H^-cyclin D1), comprising discrete CD44L and CD44H subpopulations with epithelial and mesenchymal traits, respectively^31^ (Supplementary Fig. 3a). Under basal conditions, Notch activity, as measured using *8×CSL-GFP* reporter (Fig. 2a), was highest within a subset of intermediate transitioning cells (designated as CD44T) as compared to CD44L or CD44H cells (Fig. 5a). In TGFβ-mediated EMT with a resulting increase in CD44T and CD44H cells, Notch activity was augmented further in the CD44T subpopulation (Fig. 5a), indicating that Notch activity may be augmented transiently during the CD44L-to-CD44H transition. Induction of CD44H cells may be accounted for by expansion of pre-existing CD44H cells as well as conversion from CD44L cells. Following FACS purification, CD44H cells failed to display CD44L repopulation even after extended passage (Supplementary Fig. 3b). CD44L cells that were cultured in the absence of TGFβ underwent expansion while permitting minimal CD44H cell repopulation within 5 weeks (four passages) (Supplementary Fig. 3b); however, purified CD44L cells robustly gave rise to CD44H cells upon TGFβ stimulation (Supplementary Fig. 3c; Fig. 5b). A requirement for TGFβ in CD44H cell induction was suggested as pharmacological inhibition of TGFβ receptor-mediated signaling suppressed spontaneous conversion of purified CD44L cells to CD44H cells (Supplementary Fig. 3d). A permissive role for Notch1 in CD44H cell generation via TGFβ-induced EMT was implicated further as a γ-secretase inhibitor (GSI), *DNMAML1* or *NOTCH1*-directed shRNA each individually attenuated TGFβ-mediated CD44H cell expansion from purified CD44L cells (Supplementary Fig. 3c; Fig. 5b). Ectopic ICN1 augmented CD44H cell expansion from TGFβ-stimulated purified CD44L cells where ICN1 failed to influence spontaneous CD44H cell expansion in the absence of TGFβ (Fig. 5c). Taken together, these data indicate that Notch1 activation is required, but not sufficient, for EMT-mediated CD44H cell induction in EPC2T cells.

### Transcriptional repression of Notch3 via Zeb1 permits Notch1-mediated EMT

As our findings indicate a role for Notch in EMT, we next sought to determine how Notch1 regulates cell fate in squamous epithelia. The *8×CSL-GFP* reporter activity was unaffected in CD44T and CD44H cells upon calcium-mediated squamous-cell differentiation (Fig. 5a)^4^. In CD44L cells, which displayed differentiation in organotypic 3D culture (OTC) when compared to invasive CD44H cells (Fig. 5d), calcium treatment enhanced Notch activity in a subset of cells (Fig. 5a). Thus, local environmental cues may impact Notch signaling to direct cell fate determination. In agreement with this notion, pharmacological inhibition of TGFβ signaling in purified CD44H cells induced CD44L cells with concurrent downregulation of EMT markers and *NOTCH3* upregulation (Fig. 5e, f). A trend toward increased *Notch3* expression was also detected in peeled murine esophageal epithelia with 4NQO-induced neoplastic lesions following treatment with anti-TGFβ-blocking antibody 1D11 (Supplementary Fig. 3e). Gene array analysis in EPC2T cells revealed further that ectopic ICN1 expression induced a gene expression pattern compatible with squamous-cell differentiation. However, TGFβ treatment in the context of ICN1-overexpression triggered a robust shift toward an EMT-associated gene expression signature (Fig. 5g; Supplementary Fig. 3f) (GSE37994, GSE37993). qRT-PCR analysis confirmed that ICN1 and TGFβ cooperate to induce expression of EMT regulators, including *ZEB1* and *SNAI1*, while TGFβ limited ICN1-mediated expression of *NOTCH3* as well as *IVL*, the latter a marker of terminal differentiation (Fig. 5h). Although the lack of discontinuous CD44L and CD44H cell populations in EN60 and TE11 prevented their isolation for long-term cell culture analyses, the role of Notch and TGFβ signaling in induction of CD44H cells and regulation of squamous-cell differentiation was recapitulated in TE11 cells (Supplementary Fig. 3g–i).

**Fig. 6:**
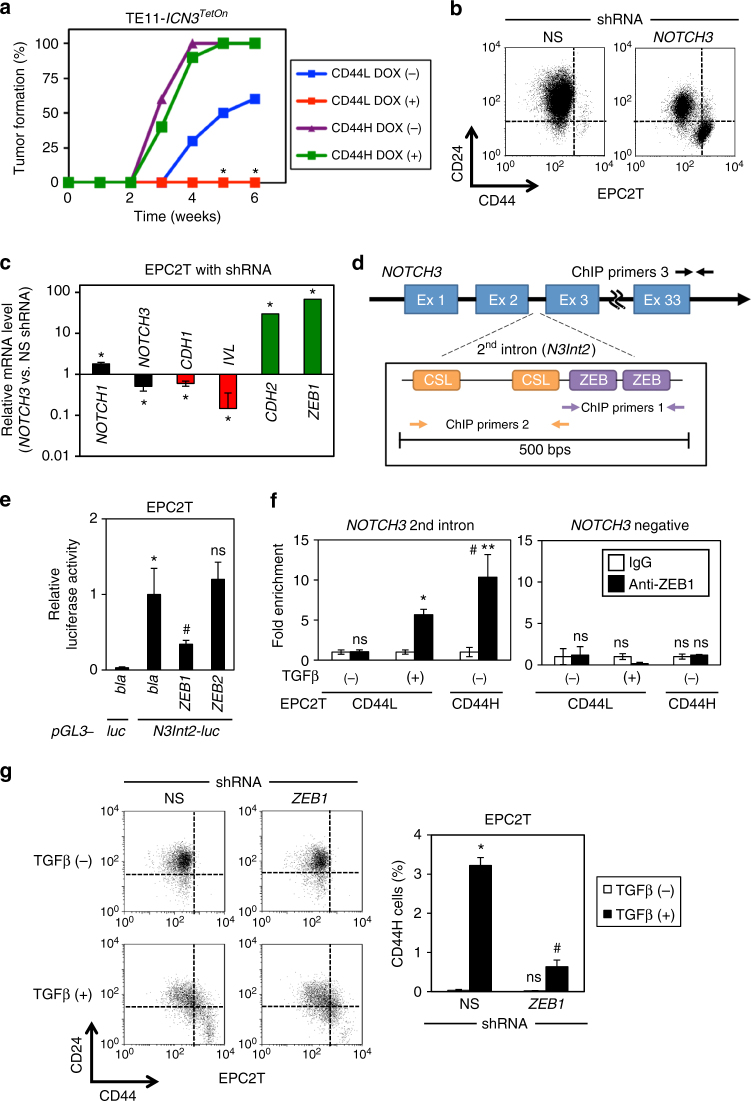
ZEB1 represses *NOTCH3*, facilitating EMT and tumor initiation. **a** Serial transplantation experiments with DOX-inducible *ICN3I-*expressing TE11 (TE11-*ICN3* ^*TetOn*^). Tumor formation rates were determined as in Fig. 2e. *P < 0.05 vs. CD44L and DOX (−); *n* = 10. **b** Representative flow cytometry scatter plots of EPC2T cells with *NOTCH3*-targeted or non-silencing (NS) control shRNA. *NOTCH3* shRNA increased CD44H cells (lower right quadrant) to 29.8 ± 0.4% s.d. as compared to 0.4 ± 0.1% s.d. in NS control (*P* < 0.0001 by Student’s *t*-test, *n* = 3). **c** qRT-PCR analysis for indicated genes comparing EPC2T cells with or without *NOTCH3* knockdown. mRNA level for each gene in NS control cells was set to 1. *P < 0.05 vs. NS, *n* = 3. **d** Schematic of *NOTCH3* second intron (*N3Int2*) region and ChIP PCR primers in **f** and Supplementary Fig. 4e. Primers amplify the region lacking ZEB1 or CSL-binding sites. **e** Transfection assays for *pGL3-N3Int2-luc* reporter activity with or without ectopic ZEB1 or ZEB2 expression. *bla*, empty vector control for ZEBs. *P < 0.05 vs. *bla* and *pGL3-luc* (empty reporter); ^#^ *P* < 0.05 vs. *bla* and *pGL3-N3Int2-luc*; ns not significant vs. *bla* and *pGL3-N3Int2-luc*; *n* = 4. **f** ChIP assays for ZEB1 binding to *N3Int2* region in purified CD44L and CD44H cells. CD44L cells were stimulated with TGFβ for 14 days to induce CD44H cells. *P < 0.0005 vs. CD44L and IgG and TGFβ (−); ^#^ *P* < 0.005 vs. CD44H and IgG and TGFβ (−); **P < 0.05 vs. CD44L and anti-ZEB1 and TGFβ (+); ns not significant vs. IgG and TGFβ (−); *n* = 3. **g** Representative flow cytometry scatter plots of EPC2T cells with *ZEB*1-targeted or NS control shRNA. *P < 0.05 vs. NS and TGFβ (−); ^#^ *P* < 0.0001 NS and TGFβ (+); ns not significant vs. NS and TGFβ (−); *n* = 3. In **b**, **g**, flow cytometry was done 7 days following lentivirus infection. All bar diagrams represent mean ± s.d. At least three independent replicates were performed for all experiments. Fisher’s exact test was used for percentage comparisons in **a**. Student’s *t*-test was used for paired data comparisons in **b**, **c**, **f**. ANOVA with Tukey’s post hoc test was used for multiple comparisons in **e**, **g**

We investigated next the mechanistic role of Notch3 in EMT in the context of ESCC. TE11 serial transplantation experiments revealed that DOX-induced ectopic ICN3 suppressed tumor initiation by CD44L cells (Fig. 6a). In EPC2T cells, ectopic ICN3 induced *IVL* in the presence or absence of TGFβ stimulation (Supplementary Fig. 4a, b). Moreover, ectopic ICN3 abrogated TGFβ-mediated CD44H cell expansion in EPC2T cells (Supplementary Fig. 4c), while *NOTCH3* knockdown was sufficient to promote CD44H cell expansion (Fig. 6b) coupled with decreased differentiation and increased EMT characteristics (Fig. 6c) in the absence of TGFβ stimulation. These findings indicate that Notch3, unlike Notch1, may limit EMT so as to permit squamous-cell differentiation. To define the mechanism through which Notch3 expression is suppressed during EMT, we analyzed the *NOTCH3* locus by the ECR browser^47^. This analysis predicts two conserved ZEB-binding sites in the *NOTCH3* second intron (*N3Int2*) adjacent to the CSL-binding sites (Fig. 6d) that is occupied by ICN1 during squamous-cell differentiation^4^. Hypothesizing that ZEB transcription factors repress *Notch3*, we evaluated the influence of ectopic ZEB1 or ZEB2 expression upon *N3Int2*-modulated transcriptional activity. ZEB1 specifically suppressed basal *pGL3-N3Int2-luc* reporter activity and also abrogated reporter activation via DOX-induced ectopic ICN1 (Fig. 6e; Supplementary Fig. 4d). Chromatin immunoprecipitation (ChIP) analysis revealed enrichment of ZEB1 binding to the *N3Int2* region in purified CD44L cells with TGFβ stimulation and purified CD44H cells without TGFβ stimulation (Fig. 6f). TGFβ did not prevent ICN1 from binding to the *N3Int2* region (Supplementary Fig. 4e), suggesting that ZEB1 binding may predominate over ICN1 to repress *NOTCH3* transcription. *ZEB1*-directed shRNA attenuated TGFβ-induced CD44H cell expansion, further implicating ZEB1 as a critical positive regulator in EMT-mediated generation of CD44H cells (Fig. 6g).

### Notch1 at SCC invasive fronts predicts poor patient prognosis

**Fig. 7:**
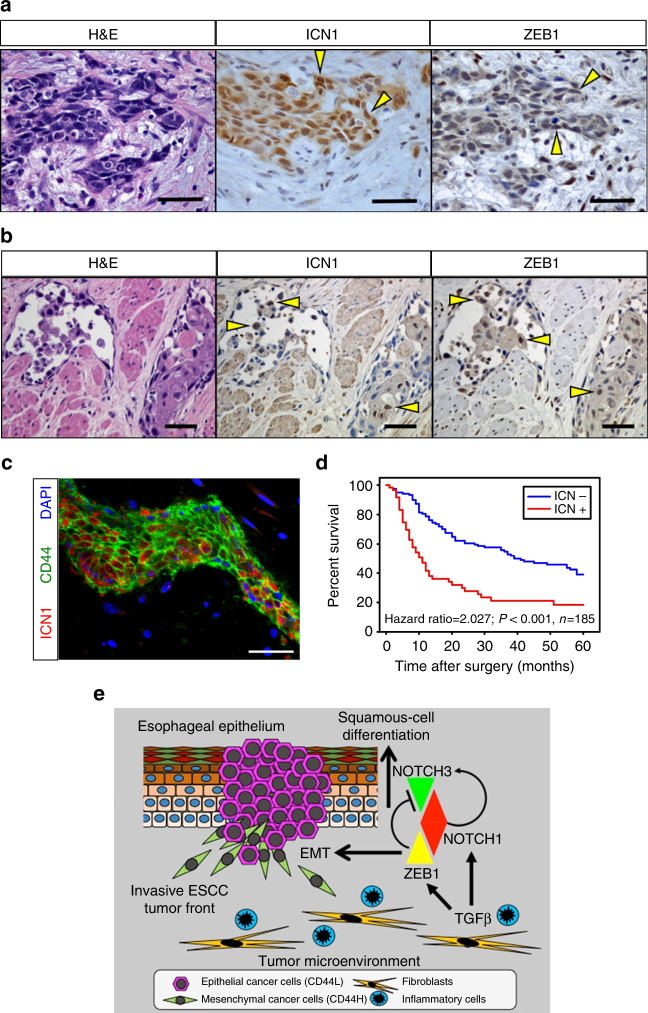
NOTCH1 activation and ZEB1 expression in invasive ESCC predicts poor prognosis. **a**, **b** Representative IHC images for ICN1 and ZEB1 in ESCC cells in a deep invasive tumor nest and ESCC cells invading into lymphatic vessels. **a** ESCC #55; **b** ESCC #62 in Supplementary Data 1. **c** Representative IF image for ICN1 and CD44 in invasive ESCC cells. Scale bars, 50 μm in **a**–**c**. **d** Survival curve for 185 post-surgical ESCC patients with or without ICN1 expression at the tumor invasive front. **e** Model of the role of Notch1 in EMT in ESCC. Notch1 activation promotes tumorigenicity and heterogeneity in SCC via EMT. Notch1 drives squamous-cell differentiation by inducing Notch3 in normal squamous epithelia as well as SCC cells. Notch3 limits EMT. In response to TGFβ from the tumor microenvironment (e.g., cancer-associated fibroblasts and inflammatory cells), however, transcriptional repression of *Notch3* by ZEB1 permits Notch1-mediated induction of CD44H cells via EMT. Notch activation may result in the generation and maintenance of CD44H cells possessing mesenchymal properties and enhanced malignant potential. EMT allows neoplastic cells to cope with stress during carcinogenesis and disease progression (e.g., genotoxic stress induced by 4NQO). CD44H cells produce pro-tumorigenic cytokines (e.g., IL-6) and tissue remodeling factors (e.g., MMP13, LOX, and POSTN) (Supplementary Fig. 3e)

To demonstrate further the importance of Notch1 activity in pathogenesis of human SCCs, we analyzed surgically procured tissue samples (Supplementary Data 1) by immunohistochemistry (IHC) with two independent NOTCH1 antibodies, both detecting nuclear NOTCH1 at a 95.24% concordance rate (*κ* = 0.664, 95% CI: 0.008–1.0, Kappa statistic, *n* = 21, Supplementary Fig. 5). ICN1 (ICN1^Val1744^) was readily detectable in normal esophageal squamous epithelia (*n* = 102) as described^4^ and was upregulated in superficial precancerous as well as early invasive ESCC lesions (Supplementary Fig. 6). Within tumors invading into submucosa or muscularis propria, most SCC cells (>80%) did not express ICN1 with or without *NOTCH1* mutations^6, 7^; however, a small subset of SCC cells expressed ICN1 in the invasive tumor front (35.3% for HNSCC, *n* = 17; 37.0% for ESCC, *n* = 227) (Fig. 7a; Supplementary Fig. 6). Accompanied by desmoplastic stroma, ICN1-positive invasive SCC often displayed spindle-shaped cell morphology and ZEB1 co-localization (17.1%, *n* = 174) (Fig. 7a; Supplementary Fig. 7) with ICN1 and ZEB1 expression being correlated (*ρ* = 0.31, *P* < 0.0001, Pearson correlation). When stratified by co-localization status, expression of ICN1 and ZEB1 was relatively uncorrelated for subjects lacking co-localization (*ρ* = −0.1, *P* = 0.2, Pearson correlation), while expression was correlated more strongly for those showing co-localization (*ρ* = 0.37, *P* < 0.05, Pearson correlation, *n* = 30). Additionally, these cells were often found invading into lymphatic vessels (Fig. 7b). NOTCH3 expression was low, if not absent, in SCC cells with concurrent ICN1 and ZEB1 expression (Supplementary Fig. 7). Moreover, ICN1 was expressed in a subset of ESCC cells with elevated CD44 expression (Fig. 7c). Evaluation of ICN1 in relation to clinicopathological data revealed that ICN1 was significantly associated with increased lymph node and distant metastases and advanced disease stages (Supplementary Table 1). Finally, analyses of clinical databases revealed that ICN1 expression at the invasive tumor front predicts independently for poor prognosis (Fig. 7d; Supplementary Table 2).

## Discussion

The current study highlights an oncogenic role for Notch1 in SCC via EMT-mediated induction of CD44H cells with enhanced malignant potential (Fig. 7e). We demonstrate that Notch1 and Notch3 may have opposing functions to allow expansion of CD44H cells both in vitro and in vivo. Moreover, we find that TGFβ guides Notch1 to drive EMT via a previously undescribed robust shift in the spectrum of Notch1 target genes including *Notch3*, which is essential in squamous-cell differentiation^4^ and senescence^12, 48^. Given the microRNA-mediated crosstalk between Notch signaling and ZEB1^33, 49^, our findings indicate that direct repression of *NOTCH3* by ZEB1 is a novel mechanism through which ZEB1 may influence Notch1-mediated cell fate determination. Activated Notch1 (i.e., ICN1) interacts with TGFβ downstream effector SMAD3^45^. ZEB1 also binds physically to SMAD3 to enhance TGFβ-mediated transcription^34^. Additionally, ZEB1 promotes tumorigenicity by repressing stemness-inhibiting microRNAs in pancreas cancer^50^, where ZEB1 induction and EMT have been implicated as the earliest event preceding tumor formation in cell-lineage tracing experiments^51^. Taken together, these preclinical studies suggest that Notch1 and ZEB1 may cooperate to promote carcinogenesis and SCC progression via TGFβ-mediated EMT and offer the TGFβ–ZEB1–Notch1 axis as potential therapeutic target in SCC.

Despite a well-established role for the Notch pathway in regulation of varied cell fates in a tissue-dependent and context-dependent manner, the molecular mechanisms governing Notch-mediated cell fate determination have yet to be fully elucidated. In non-transformed esophageal epithelium, Notch1-dependent transcriptional upregulation of *Notch3* mediates squamous-cell differentiation as shown by us^4^. In the current study, we find Notch1 facilitates EMT-mediated expansion of CD44H cells with enhanced malignant potential. This raises the question of how Notch1 may act as a signal to induce both differentiation and dedifferentiation (i.e., EMT) in squamous epithelium. One potential explanation for this dichotomy is the influence of tissue microenvironment upon Notch-mediated cell fate determination. Indeed, microarray gene expression analysis in ICN1-overexpressing transformed esophageal keratinocytes indicates that TGFβ induces a dramatic shift in the spectrum of Notch1 target genes from a profile enriched for genes involved in squamous-cell differentiation to one consistent with EMT. Notch1 may also facilitate senescence in response to TGFβ in esophageal keratinocytes with intact cell cycle checkpoint functions^12^. The complexity in the role of Notch signaling in tumor biology is echoed by that of TGFβ as intratumoral cancer cell heterogeneity represents cancer cells that can respond to TGFβ and those cannot, the latter emerging during disease progression^52^. Thus, like TGFβ, Notch1 may have differential roles in cancer initiation and development. Besides TGFβ, hypoxia and inflammation may activate Notch1 to promote EMT in the tumor microenvironment via transcriptional factors such as HIF1α^17^ and NF-κB^53^, respectively. Potential influence of these factors upon Notch1-mediated cell fates warrants further investigation in SCC as well as squamous epithelia under eosinophilic esophagitis where EMT and Notch3 downregulation are implicated (P.M.C. and H.N., unpublished observation).

While our own published findings and those of others have implicated Notch1 as a tumor promoter in SCCs^12, 54–56^, the current study is the first to demonstrate in vivo that Notch1 facilitates tumor initiation by converting CD44L cells to highly tumorigenic CD44H cells with mesenchymal traits, complementing the earlier studies implicating EMT and ZEB1 in the CD44L-to-CD44H transition^24, 28, 57^. While Notch1 activation and EMT were detected at the invasive tumor front, SCC cells with epithelial characteristics were commonly present in xenograft tumors, 4NQO-induced tumors, and human SCC samples. In our cell-lineage tracing experiments utilizing *K5Cre* ^*ERT2*^ *;R26tdTomato* ^*lsl/lsl*^ mice carrying 4NQO-induced tumors, complete loss of epithelial characteristics was uncommon, albeit present (Supplementary Fig. 1d). Despite enhanced expression of mesenchymal-associated genes in EpCAM^neg^ cells (Fig. 1c, d), most tumors displayed a broad range of continuous EpCAM expression (Supplementary Fig. 1c), suggesting a highly heterogeneous and plastic nature of SCC cells. Besides squamous-cell differentiation, Notch3 may be permissive for mesenchymal–epithelial transition or epithelial–mesenchymal hybrid characteristics of cancer cells by limiting EMT^58, 59^. These findings have potential clinical impact as reducing CD44H cells may delay disease progression or post-therapeutic recurrence and increase sensitivity to chemotherapeutic agents, providing a platform for new human clinical trials. Given the interplay between Notch1 and Notch3 in regulating esophageal cell fate, the use of pan-Notch inhibitors may prove problematic. Indeed, genetic Notch inhibition via DNMAML1 expression accelerates carcinogen-mediated esophageal tumor initiation, growth and invasion in vivo^60, 61^. Tumor initiation by CD44H cells may be independent of Notch because purified CD44H cells gave rise to tumors with or without DOX-induced ICN1 expression or in the presence of DNMAML1 (Fig. 3c). Interestingly, Notch inhibition by DNMAML1 permits clonal immortalization and expansion of murine esophageal epithelial cells^61^. Thus, Notch1 activation is essential for the induction of CD44H cells while it may be dispensable for their maintenance and tumor initiation by CD44H cells. Such a premise is corroborated by ICN1 expression localized to tumor invasive fronts, but not in the majority of expanding ESCC cell populations (Supplementary Fig. 6). Notch signaling can be selectively modulated by Notch receptor paralog-specific antibodies^62, 63^. TGFβ signaling can be also regulated by TGFβ inhibitors^64^. Notably, the observed inhibitory effects of anti-TGFβ antibody upon tumor progression in mice (Fig. 4) may involve TGFβ downstream effectors other than Notch1, representing a limitation of the current study. Since TGFβ-induced EMT involves autophagy in ESCC cells where pharmacological inhibition of autophagy flux by chloroquine decreases CD44H cells, an alternative approach to target EMT and CD44H cells^30^. Future studies evaluating the efficacy of these agents in SCC therapy are currently underway in preclinical models.

Technical innovation in this study includes use of a HNSCC PDX model as well as a cell-lineage tractable mouse model of ESCC through which EMT was for the first time documented unequivocally in SCC cells in situ. Additionally, we have developed an esophageal 3D organoid platform that recapitulates esophageal tissue architecture ex vivo and can be utilized to model esophagitis^65^. Here, we apply this model system to SCC, generating murine ESCC-derived 3D organoids for the first time (Fig. 1e). Upon ex vivo culture, single-cell-derived 3D organoids from 4NQO-induced dysplasia and ESCC tumors display morphological characteristics of neoplastic epithelium, including nuclear atypia, perturbed squamous-cell differentiation and evidence of EMT, which are maintained upon organoid passaging (K.A.W., P.M.C., and H.N., unpublished observation). We have also successfully performed ex vivo Cre-mediated recombination in 3D organoids generated from neoplastic esophageal keratinocytes of 4NQO-treated *Notch1* ^*loxP/loxP*^ mice, demonstrating that Notch1 is required for organoid formation and EMT-like features in neoplastic 3D organoids (Figs. 2f, 3e). *Notch1* deletion in the murine skin promotes SCC development in a non-cell autonomous fashion due to inflammation associated with epidermal barrier defects^5^, limiting the assessment of the cell-autonomous oncogenic role of Notch1 in vivo. The 3D organoid system provides insights about cell-autonomous oncogenic functions of Notch1 in the absence of inflammatory milieu ex vivo, complementing in vivo experiments. The 4NQO model requires a long-term 4NQO administration (16 weeks) where tumors arise 6–9 weeks after 4NQO withdrawal during the observation period; however, it is not precisely known when malignant transformation occurs in this model. The single-cell-derived 3D organoids have a potential to detect neoplastic changes in a more sensitive and quantitative manner than conventional morphological tissue assessment. Such a study is underway. Once an appropriate window of 4NQO-induced malignant transformation is determined, our cell-lineage traceable mice can be utilized for genetic ablation of *Notch1* following 4NQO administration to elucidate how *Notch1* may exert its oncogenic role by promoting EMT and tumor initiation and/or progression in vivo. Alternatively, such mice may be treated with Notch1-specific antagonistic antibody^63^ or anti-Notch3 agonistic antibody^62^, the latter can be used for targeted activation of Notch3. While these findings identify 3D neoplastic organoids as sustainable resource for functional and mechanistic investigations into the biology of ESCC, it remains to be determined how faithfully organoid genetics and biology mimic that of the tissue from which they are derived upon extended ex vivo culture. Ongoing studies include procuring of a bank of human patient-derived ESCC 3D organoids that may be utilized as an experimental platform for discovery and validation of novel translational applications for prognosis and therapy in the setting of personalized medicine.

Taken together, the current study supports a tumor-promoting role of Notch1 in SCC while also providing novel mechanistic insight into how the local tissue microenvironment may influence Notch-mediated cell fate determination.

## Methods

### Patients and tissue samples

Surgically removed tissue samples (Supplementary Data 1) were described previously^11, 66–68^ or newly procured at Kagoshima University Hospital in accordance with Institutional Review Board standards and guidelines. Informed consent was obtained from all human subjects. Most samples were available as tissue microarrays (TMAs)^67, 68^ containing primary ESCC (*n* = 171), carcinoma in situ (CIS; *n* = 9), IEN (*n* = 7), and normal mucosa (*n* = 114). Samples with *NOTCH1* mutations (*n* = 4; E755*, D469Y, R1279D, and D1457G) and wild-type *NOTCH1* (*n* = 15) were identified by DNA sequencing (SRP072948)^69^. Given the limitation of TMAs to assess intratumoral cancer cell heterogeneity^70^, we examined whole paraffin blocks (*n* = 244) by Hematoxylin and Eosin (H&E) staining and IHC, as described below, following preliminary analysis with TMAs. Survival analysis was done on IHC data for 185 ESCC patients who did not receive chemotherapy or radiation therapy prior to surgery.

### Esophageal epithelial cell-lineage traceable mice and 4NQO treatment

The *K5Cre* ^*ERT2*^ transgenic mouse strain^68^ was intercrossed with *R26tdTomato* ^*lsl/lsl*^ (Jackson Laboratory, Bar Harbor, ME) carrying the homozygous Rosa26 locus with knocked-in *tdTomato* fluorescent protein as a reporter under the loxP-stop-loxP sequence. The resulting *K5Cre* ^*ERT2*^ *;R26tdTomato* ^*lsl/wt*^ mice were crossed with *R26tdTomato* ^*lsl/lsl*^ mice to generate *K5Cre* ^*ERT2*^;*R26tdTomato* ^*lsl/lsl*^ mice. Similarly, *K5Cre* ^*ERT2*^ *;p53* ^*loxP/loxP*^ mice were generated with *p53* ^*loxP/loxP*^ mice (Jackson Laboratory). *R26tdTomato* ^*lsl/lsl*^ mice were also crossed with *p53* ^*loxP/loxP*^ to generate *R26tdTomato* ^*lsl/wt*^ *;p53* ^*loxP/wt*^ mice, which were then crossed to generate *R26tdTomato* ^*lsl/lsl*^ *;p53* ^*loxP/loxP*^ mice. The *K5Cre* ^*ERT2*^ *;p53* ^*loxP/loxP*^ mice were further crossed with *R26tdTomato* ^*lsl/lsl*^ *;p53* ^*loxP/loxP*^ mice to generate *K5Cre* ^*ERT2*^ *;R26tdTomato* ^*lsl/wt*^ *;p53* ^*loxP/loxP*^. These mice were further crossed with *R26tdTomato* ^*lsl/lsl*^ *;p53* ^*loxP/loxP*^ to generate and maintain the *K5Cre* ^*ERT2*^ *;R26tdTomato* ^*lsl/lsl*^ *;p53* ^*loxP/loxP*^ strain. The *L2Cre* transgenic mouse strain^71^ was intercrossed with *p53* ^*R172H/R172H*^ mice^72^ to generate *L2Cre;p53* ^*R172H/wt*^ mice.

We administered TAM (Sigma-Aldrich, St. Louis, MO; 0.25 mg/g body weight) via oral gavage to 3–4-month-old *K5Cre* ^*ERT2*^ *;R26tdTomato* ^*lsl/lsl*^ and *K5Cre* ^*ERT2*^ *;R26tdTomato* ^*lsl/lsl*^; *p53* ^*loxP/loxP*^ littermates 2 weeks before starting 4-Nitroquinoline *N*-oxide (4NQO) (Sigma-Aldrich) treatment. Mice received 100 µg/ml 4NQO in 2% propylene glycol (MP Biomedicals, Solon, OH) in drinking water for 16 weeks and were followed up for 8–10 weeks (or earlier if mice were ill) after 4NQO withdrawal as described^73^. To inhibit TGFβ receptor-mediated signaling, mice were treated for 2 weeks with either anti-TGFβ antibody 1D11 (intra-peritoneally), neutralizing all three isoforms of TGFβ^46^ (a gift of Dr. Singhal, University of Pennsylvania; 3 mg/kg, three times per week) or control IgG (intra-peritoneally) with injections initiated 4 weeks following 4NQO withdrawal. At the time of sacrifice, one half of the dissected esophagus or a tumor (if visible macroscopically) from each mouse was fixed in 4% paraformaldehyde and paraffin-embedded for morphological analyses. The other half of esophagi and tumors was dissociated as described previously^74^ and cell suspensions were subjected to flow cytometry or FACS and organoid formation assays. All experiments were done under approved protocols from the University of Pennsylvania Institutional Animal Care and Use Committee (IACUC). Sample size for groups were projected based on data from pilot studies. Animals were only excluded from analyses in event of death from procedure-related causes (e.g., death following oral gavage) that were unrelated to experimental differences between groups. Animals were randomized to treatment groups with consideration given to representation of both sexes. Investigators were informed of groups during treatment phase of experiments. Upon processing, tissues were given a unique identifier to blind investigators during analyses and outcome assessments.

### Xenograft transplantation experiments

Xenograft transplantation experiments were done as described^11, 12^. In brief, 1–5 × 10^6^ cells were suspended in 50% Matrigel and implanted subcutaneously into the dorsal skin of female athymic *nu*/*nu* mice (4–6 weeks old; Charles River Breeding Laboratories). PDX tumor transplantation was performed as previously described with fragments of passaged PDX tumors implanted under the dorsal skin of NOD/SCID/IL2 receptor γ-chain-deficient (NSG) mice^75^. Tumor growth was monitored and mice were sacrificed 6–10 weeks after inoculation for flow cytometry and histopathological analyses. One half of each tumor was fixed in formalin and embedded in paraffin blocks. The other half of each tumor was enzymatically dissociated and subjected to flow cytometry and FACS for serial transplantation experiments where 10–10^3^ cells were injected into recipient mice to determine tumor formation rates. For tetracycline/DOX-inducible (TetOn) transgene expression in xenograft tumors, mice were fed daily with DOX-containing pellets (20 mg/kg). To inhibit TGFβ receptor-mediated signaling, mice were treated for 2 weeks with either anti-TGFβ antibody 1D11 (intra-peritoneally) (3 mg/kg, 2–3 times per week) or control IgG (intra-peritoneally), beginning when tumor volume reached 60 mm^3^. All experiments were approved by the University of Pennsylvania IACUC or under Wistar Institute IACUC protocols 112652/112655. Sample size for groups were projected based on previous xenograft studies then adjusted following acquisition of data in initial experiment. Animals were only excluded from analyses in event of death from procedure-related causes (e.g., sepsis) that were unrelated to experimental differences between groups. Animals were randomized by cage upon arrival. Investigators were informed of groups during treatment phase of experiments. Upon processing, tumors were given a unique identifier to blind investigators during analyses and outcome assessments.

### DNA constructs

A lentiviral vector pTRIPZ expressing DOX-inducible shRNA directed against human *NOTCH1* (Notch1-A; V2LHS_149557 and Notch1-B; V3LHS_637133) and pTRIPZ-NS carrying a non-silencing scrambled sequence (RHS4346) (GE Dharmacon, Lafayette, CO) were purchased. Other shRNA sequences in pGIPZ (GE Dharmacon) targeting *NOTCH3* (Notch3-A; V2LHS_229748 and Notch3-B; V2LHS_93017), *ZEB1* (ZEB1-A; V2LHS_116663 and ZEB1-B; V2LHS_116659), and a non-silencing scrambled sequence (RHS4348) were used as described previously^11, 12, 19^.

To generate a lentiviral vector pTR-tRFP expressing constitutively *turbo RFP* (*tRFP*) under ubiquitin C (UBC) promoter, a DOX-inducible gene expression vector pTRIPZ-MCS^12^ was modified by removal of *Bam*HI and LoxP sites and a subsequent insertion of new multiple cloning sites (MCS) comprising 5′-*Age*I-*Bam*HI-*Sna*BI-*Eco*RI-*Xho*I-*Mlu*I-3′, resulting in the creation of pTRIPZ-MCS2. We then amplified an open reading frame (ORF) for *turbo RFP* (*tRFP*) by PCR using pTRIPZ-NS (RHS4743, GE Dharmacon) as a template with primers 5′-AGCGCTAGCGCCACCATGAGCGAGCTGATC-3′ and 5′-AGCGCGGCCGCTTATCTGTGCCCCAGTTTGCTAGG-3′. Following removal of rtTA-encoding ORF flanked by *Nhe*I and *Not*I sites, the purified *tRFP* ORF was subcloned into the *Nhe*I and *Not*I sites of pTRIPZ-MCS2, resulting in creation of pTR-tRFP. To generate a lentiviral vector carrying *8×CSL-GFP* reporter, an ORF for *GFP* was isolated from pBABE-zeo-GFP^19^ and ligated into the *EcoR*I and *Xho*I sites of pTR-tRFP, generating pTR-tRFP-GFP. A DNA fragment containing a concatemer of eight copies of CSL-binding sites fused to minimal SV40 (mSV40) promoter was isolated from 8×*CSL-luc* ^76^ by PCR with primers 5′-AGCTCTAGACTCTTACGCGT GCTAGCTCGA-3′ and 5′-AGCACCGGTTTTGCAAAAGCCTAG GCCTCCA-3′ to replace the sequence containing tetracycline response elements and minimal CMV promoter from pTR-RFP-GFP at the *Xba*I and *Age*I sites, resulting in a creation of pTR-RFP-8×CSL-GFP.

To delete human *NOTCH1* via Crispr/Cas9-mediated gene editing, five candidate guide RNA (gRNA) sequences were subcloned into lentiCRISPR v1 (gift of a gift from Feng Zhang; Addgene, plasmid #52961) at its *Bsm*BI sites and used as described^77^. gRNAs 1, 2, and 5 targeting chromosome 9 *NOTCH1* exon 14 (EGF-like region at 139,407,894–139,407,913) or exon 19 (EGF-like region at 139,403,465–139,403,484) and exon 34 (PEST domain at 139,390,539–139,390,558), respectively, were found to be equally effective to delete *NOTCH1*.

To generate a retroviral vector BABE-zeo-FLAG-DNMAML1, we amplified an Flag-MAML1^1–302^ ORF by PCR using pFLAG-CMV2-MAML1 (1–302) (a gift from Dr. Wu, University of Florida) as a template with primers 5′-GCACGGATCCGCCACCATGGACTACAAAGACGATGA CGACAAG-3′ and 5′-GCACGTCGACCTAGAATTCCGTCTTAATATTAATGTCCTGTGCC-3′ and subcloned at the *Bam*HI and *Sal*I sites of pBABE-zeo^78^. To generate pBABE-bla-ZEB1 and pBABE-bla-ZEB2, ZEB1 and ZEB2 ORFs in pCR-BluntII-TOPO were purchased from GE Dharmacon (clone 40036600 for ZEB1 and clone 40124124 for ZEB2). The ZEB1 and ZEB2 ORFs were amplified by PCR with primers 5′-CGCTGGATCCGCCGCCACCATGGCGGATGG CCCCAGGTG-3′ and 5′-CGGAGTCGACTTAGGCTTCATTTGTCTTTTC-3′ for ZEB1 and 5′-CG CTGGATCCGCCGCCACCATGAAGCAGCCGATCATGGC-3′ and 5′-CGGAGTCGACTTACATGCCATCTTCCATATTG-3′ for ZEB2, respectively, and subcloned at the *Bam*HI and *Sal*I sites of pBABE-bla^79^.

The 2nd intron of *NOTCH3* (*N3Int2*) containing ZEB1-binding sites (488-bp) was amplified by PCR using the input DNA sample from ChIP assay as a template with primers 5′-GGGAGATCTGGTTCCCACGCTCTGCGTCC-3′ and 5′-GGGCTCGAGCCGCGCCTGGAATACTGCCG-3′. Then the amplified DNA was subcloned at the *Xho*I and *Bgl*II sites of pGL3 promoter (Promega, Madison, WI), creating a plasmid pGL3-N3Int2-luc. All constructs were verified by DNA sequencing.

### Cell culture, genetic modifications, and pharmacological treatments of cells

TE11, EN60, EPC2T, and derivatives were grown at 37 °C in a 5% CO_2_ atmosphere as described previously^12^. Additional genetic modifications were done via stable gene transfer of the above retroviral and lentiviral constructs as described previously^12^. Stable cell lines were established by drug selection for 7 days with 1 µg/ml of Puromycin (Invitrogen) for genes transduced via pTRIPZ or lentiCRISPR; and 0.5 µg/ml zeocin (Invitrogen) for Flag-MAML1^1–302^ transduced via pBABE-zeo. Cells transduced with GFP (pGIPZ) or tRFP (pTR-tRFP) were selected for the brightest level of fluorescence (top 20%) by FACS. EPC2T, EN60, and derivatives were kept undifferentiated in Keratinocyte serum-free medium (Invitrogen, Carlsbad, CA) containing a low concentration (0.09 mM) of CaCl_2_. Squamous-cell differentiation was induced by 0.6 mM CaCl_2_ (Sigma-Aldrich) as described^4^. Compound E (Calbiochem, La Jolla, CA), a GSI, and SB431542 (Calbiochem), a TGFβ type I receptor kinase inhibitor were reconstituted in dimethyl sulfoxide (DMSO) (Sigma-Aldrich). Cells were treated with 1 µM Compound E or 5 ng/ml recombinant human TGFβ1 (R&D Systems, Minneapolis, MN) as optimized previously^4, 19, 80^. SB431542 was used at 10 µM. Dox was used at 1 µg/ml for DOX-inducible transgene expression^12^. Phase contrast images were acquired using a Nikon Eclipse E600 microscope. EPC2-hTERT cells and derivatives were established and extensively characterized by us^12, 30, 74^. TE11^12, 30, 81^ and EN60^12, 82^ cells were generous gifts from Dr. Tetsuro Nishihira (Tohuko University, Sendai, Japan) and Dr Hiroshi Shirasawa (Chiba University, Chiba, Japan), respectively, and were extensively characterized by us^12, 30, 74, 83^. The earliest frozen stocks of all cell lines have been stored at the Cell Culture Core of the University of Pennsylvania. We have propagated cells from frozen stocks of original vials that were authenticated by short tandem repeat profiling (ATCC) for highly polymorphic microsatellites to validate the identity of cells by comparing cells at the earliest stocks and those grown >8–12 passages. All cell lines undergo routine mycoplasma testing.

### Organotypic 3D culture

OTC was carried out as described previously^74^. In brief, 0.5 × 10^6^ of epithelial cells were seeded on top of the collagen/Matrigel matrices containing FEF3 human fetal esophageal fibroblasts, and grown in submerged conditions for 4 days. Cultures were then raised to the air–liquid interface for additional 4 days and harvested for morphological assessment. Each OTC experiment was performed in triplicate.

### Ex vivo esophageal organoid 3D culture

Esophageal keratinocytes were isolated from vehicle-treated or 4NQO-treated *K5Cre* ^*ERT2*^ *;R26tdTomato* ^*lsl/lsl*^ or *Notch1* ^*loxP/loxP*^ (Jacskon Laboratories) mice under an IACUC-approved protocol as described previously^65, 84, 85^. Using 24-well plates, 5000 cells were seeded per well in 50 μl Matrigel. After solidification, 500 μl of DMEM/F12 supplemented with 1× Glutamax, 1× HEPES, 1× N2 Supplement, 1× B27 Supplement, 0.1 mM *N*-acetyl-L-cysteine (Sigma-Aldrich), 50 ng/ml mouse recombinant EGF (R&D Systems), 2.0% Noggin/R-Spondin-conditioned media and 10 μM Y27632 (Tocris Biosciences, Bristol, UK) were added and replenished every other day. For ex vivo recombination, organoids were cultured in the presence of Adenovirus vector containing Cre recombinase and GFP (University of Iowa Gene Transfer Vector Core). Adenovirus vector containing GFP alone was used as a control. Adenovirus vectors were used at 1:500 at the time of organoid plating. Organoid formation rate was calculated as the percentage of the number of organoids formed at day 7 per total number of cells seeded at day 0. After 14 days organoids were recovered by digesting Matrigel with Dispase I (BD Biosciences, San Jose, CA; 1 U/ml) and fixed overnight in 4.0% paraformaldehyde. Specimens were embedded in 2.0% Bacto-Agar: 2.5% gelatin prior to paraffin embedding.

### RNA isolation, cDNA synthesis, qRT-PCR, and microarray analyses

RNA isolation, cDNA synthesis, and qRT-PCR were done using StepOnePlus™ Real-Time PCR System (Applied Biosystems) by TaqMan^®^ Gene Expression Assays (Applied Biosystems) for *NOTCH1* (Hs01062014_m1), *Notch1* (Mm00435249_m1), *NOTCH3* (Hs00166432_m1), *Notch3* (Mm00435270_m1), *IVL* (Hs00846307_s1), *CK13* (s00999762_m1), *CDH1* (Hs00170423_m1), *CDH2* (Hs00983062 _m1), *ZEB1* (Hs00232783_m1), *SNAI1* (Hs00195591_m1), and *PAI1* (Hs01126606_m1), and SYBR^®^ Green PCR for human *ACTB* (β-Actin) as well as murine *Cdh1* (5′-TCAAGCTCGCGGATAACCAGAACA-3′ and 5′-ATTCCCGCCTTCATGCAGTTGTTG-3′), *Cdh2* (5′-ATGGCCTTTCAAACACAGCCACAG-3′ and 5′-ACAATGACGTCCACCCTGTTCTCA-3′), *Zeb1* (5′-TGAGCACACAGGTAAGAGGCC-3′ and 5′-GGCTTTTCCCCAGAGTGCA-3′), *Zeb2* (5′-TGATAGCCTTGCAAACCCTCTGGA-3′ and 5′-TCCTTCATTTCTTCTGGACCGGCT-3′), *Twist* (5′-AGCTGAGCAAGATTCAGACCCTCA-3′ and 5′-TGCAGCTTGCCATCTTGGAGT-3′), and *Gapdh* (5′-GGTGGTCTCCTCTGACTTCAACA-3′ and 5′-GTTGCTGTAGCCAAATTCGTTGT-3′) and as described^4, 11, 19^. All PCR reactions were performed in triplicate. The relative level of each mRNA was normalized to *ACTB* (β-actin) for human genes and *Gapdh* for murine genes as internal controls. Gene array experiments were done using an Affymetrix gene chip (U133+v2.0) (Affymetrix, Santa Clara, CA) as described^11, 31^. Data were deposited at the NCBI Gene Expression Omnibus (http://www.ncbi.nlm.nih.gov/geo) (accession #GSE37994).

### Immunoblot analysis

Whole cell lysates were prepared as described^4, 11^. 20 μg of denatured protein was fractionated on a NuPAGE Bis-Tris 4–12% gel (Invitrogen). Following electrotransfer, Immobilon-P membranes (Millipore) were incubated with primary antibodies for rat monoclonal anti-NOTCH1 5B5 (1:1000; 3447, Cell Signaling, Danvers, MA), rabbit monoclonal anti-ICN1^Val1744^ D3B8 (1:1000; 4147, Cell Signaling), rat monoclonal anti-NOTCH3 8G5 (1:1000; 3446, Cell Signaling), mouse monoclonal anti-E-cadherin (1:10,000; 610182, BD Biosciences), mouse monoclonal anti-N-cadherin clone 32 (1:1000; 610920, BD Biosciences), mouse monoclonal anti-Involucrin clone SY5 (1:1000, I9018, Sigma-Aldrich) and mouse monoclonal anti-β-actin (1:10,000; AC-74, Sigma-Aldrich), and then with the appropriate HRP-conjugated secondary antibody (GE Healthcare, Piscataway, NJ). β-actin served as a loading control. Uncropped images of blots from primary figures are shown in Supplementary Fig. 8.

### Flow cytometry and FACS

Flow cytometry and FACS were performed as described previously^86^. FACSCalibur or LSRII (BD Biosciences, San Jose, CA) and FlowJo (Tree Star, Ashland, OR) were used for flow cytometry. FACS Vantage SE and FACS Aria II (BD Biosciences) were used to sort purified CD44^high^-CD24^low/−^ cells (CD44H) and CD44^low/−^-CD24^high^ cells (CD44L) from EPC2T, TE11 and EN60 cells, and/or xenograft tumors. Cells were suspended in Hank’s balanced salt solution (Invitrogen) containing 1% BSA (Sigma-Aldrich) and stained with the following antibodies on ice for 30 min: human PE/Cy7-anti-CD24 clone [ML5] (1:40; 311,120, BioLegend, San Diego, CA), human APC-anti-CD44 clone G44-26 (1:20; 559,942, BD Biosciences), human FITC-anti-CD326 (EpCAM) clone HEA-125 (1:50; 130-089-113, Miltenyi Biotec, San Diego, CA) and/or mouse APC-Cy7-anti-CD326 (EpCAM) Clone G8.8 (1:50; 118,217, BioLegend). 4′,6-diamidino-2-phenylindole (DAPI; 2 μg/μl; Invitrogen) was used to assess viability. CD44T cells were defined as CD44^high^-CD24^High^. To purify CD44L and CD44H cells from TE11 and EN60 xenograft tumors, tumors were minced into 1 mm^3^ pieces and incubated in Dulbecco’s Modified Eagle Medium (DMEM; Invitrogen) containing 1 mg/ml collagenase I (Sigma-Aldrich) at 37 °C for 90 min. Following centrifugation, residual tissue pieces were digested in 0.05% trypsin-EDTA (Invitrogen) at 37 °C for 10 min and then with 1 U/ml Dispase (BD Biosciences) and 100 μg/ml DNase I (#10104159001, Roche) at 37 °C for 10 min. Dissociated tumor cells were filtered with a 40 µm cell strainer (BD Biosciences) and washed prior to incubation with antibodies. Cancer cells were distinguished from host-derived stromal cells by tRFP expression detected concurrently. In 8×CSL-GFP reporter assays, tRFP-positivity was used to identify human cells while GFP expression was monitored to evaluate Notch activity. In tissue from 4NQO-treated mice and controls, tdTomato expression was used to isolate epithelial cells that had undergone *K5Cre* ^*ERT2*^-mediated recombination following TAM treatment. Following dissection, esophagi were incubated with 1 U/ml Dispase for 5 min at 37 °C then epithelial layer was peeled from underlying stroma using fine tipped forceps. Epithelia were then incubated in 0.25% trypsin two times for 5 min at 37 °C with agitation to liberate keratinocytes. Trypsin was quenched with soybean trypsin inhibitor then liberated cells were filtered with a 40 µm cell strainer prior to staining. Flow cytometry was repeated for each genotype and condition at least three times.

### Immunofluorescence (IF) and IHC

IF and IHC for NOTCH1, NOTCH3, ZEB1, E-cadherin, CD44, and tdTomato were done as described previously^4, 11, 12^. IHC for Notch1 was done with two independent anti-NOTCH1 antibodies, namely, polyclonal rabbit polyclonal anti-NOTCH1 (ab27526; Abcam; 1:250) and rabbit polyclonal anti-ICN1^V1744^ (2421; Cell Signaling; 1:100), both detecting cytoplasmic and nuclear expression of NOTCH1. The following primary antibodies were used for IHC and/or IF: rabbit polyclonal anti-NOTCH3 (ab23426; Abcam; 1:250), rabbit polyclonal anti-ZEB1 (a generous gift from Dr. Darling, University of Louisville; 1:100), mouse monoclonal anti-E-cadherin (610182; BD Biosciences; 1:100), rabbit polyclonal anti-RFP (ab34771, Abcam; 1:100). IHC signals were developed using the diaminobenzidine substrate kit (Vector Laboratories, Burlingame, CA) following incubation with secondary anti-mouse IgG (Vector; 1:600 at 37 °C for 30 min) or anti-rabbit IgG (Vector; 1:600 at 37 °C for 30 min), and counterstained with Hematoxylin (Fisher Scientific CS401-1D). For IF, Cyanine Cy2-conjugated or Cyanine Cy3-conjugated affinity-purified anti-mouse or anti-rabbit IgG (Jackson Immuno-Research; 1:600) was used for signal detection by incubating at 37 °C for 30 min, and cell nuclei were counterstained by DAPI (Invitrogen; 1:10,000). Stained objects were examined with a Nikon E600 microscope and imaged with a digital camera. The staining was assessed independently by pathologists (S.N. and A.K.S.), and the intensity was expressed as negative (0), marginally positive (0.5), weakly positive (1), moderately positive (2), or strongly positive (3). Both antibodies detect cytoplasmic and nuclear NOTCH1 at high concordance rates, although anti-ICN1^V1744^ antibody was more sensitive than ab27526 to detect nuclear Notch1 expression (Supplementary Fig. 5). The ab27526 antibody recognizes a full-length NOTCH1 expressed on the cell surface in normal squamous epithelia^4^ as well as well-differentiated SCC cells forming keratin pearl^11^. In this study, we have focused on cytoplasmic and nuclear intracellular Notch1 (ICN1) detected by anti-ICN1^V1744^.

### Transient transfection and dual-luciferase assays

Transient transfection of reporter plasmids and luciferase assays was performed as described previously^4, 80^. 1 × 10^5^ cells were seeded per well in 24-well plates 24 h before transfection. Lipofectamine^TM^ LTX and Plus^TM^ reagents (Invitrogen) were used for DNA transfection, according to the manufacturer’s instructions. 400 ng of pGL3p-N3Int2 was transfected along with or without 400 ng of pBABE-bla-ZEB1, pBABE-bla-ZEB2 of pBABE-bla (empty vector control). 5 ng of phRL-SV40-renilla luciferase vector (Promega) was co-transfected to calibrate the variation of transfection efficiencies among wells. Cells were incubated in the presence or absence of 1 µg/ml DOX to induce ICN1 in cells expressing *ICN1* ^*TetOn*^ for 48 h before cell lysis. Alternatively, 5 ng/ml TGFβ1 was added at 24 h after transfection and incubated for an additional 48 h before cell lysis. Luciferase activities were determined using Dual-Luciferase^TM^ Reporter Assay system (Promega) and ORION Microplate Luminometer (Berthold Detection Systems, USA, Oak Ridge, TN). The mean of firefly luciferase activity was normalized with the co-transfected renilla luciferase activity. Transfection was carried out at least three times, and variation between experiments was not greater than 15%.

### ChIP assay

1 × 10^7^ cells were treated with 1% formaldehyde for 10 min at 37 °C and quenched with 0.125 M glycine for 5 min at room temperature. Cross-linked chromatin was sheared into ~500 bp DNA fragments with Branson Sonifier 250 (Branson, Danbury, CT, USA) and subjected to immunoprecipitation with 2 µg/10^6^ cells with antibody for ZEB1 (sc10572, Santa Cruz), NOTCH1 (sc6014-R, Santa Cruz) or either goat IgG (sc2028, Santa Cruz) or rabbit IgG (sc2027, Santa Cruz) as negative controls. DNA was purified by QIAquick PCR purification kit (QIAGEN, Valencia, CA) and analyzed by real-time PCR using StepOnePlus™ Real-Time PCR System (Applied Biosystems, Carlsbad, CA). The following primers were used for real-time qPCR: 5′-CCCACAGCCCAACTCGGAGG-3′ and 5′-CCGCGCCTGGAATACTGCCG-3′ for ZEB1-binding sites at the 2nd intron of *NOTCH3*, 5′-GCTGGGCGCCGAGGATAG-3′ and 5′-AGACCTCGTCCCCATCTCCTAGTC-3′ for CSL-binding sites^4^ at the 2nd intron of *NOTCH3*, 5′-TGCCAAAAGAGGAAGCATAAGTA-3′ and 5′-TCAAAATCCCTGTGTAGCTGAAT-3′ for an off-target control^4^ for *NOTCHotch3*. 5′-CCCTTTCTGATCCCAGGTCT-3′ and 5′-GACCTGCACGGTTCTGATTC-3′ for a ZEB1-binding site on the of *CDH1* promoter^87^, 5′-GAAGTGGCTCCAGTGCTCAAA-3′ and 5′-ATGGCAGTGCATGCCTGTAGT-3′ for an off target control for *CDH1* promoter^87^, 5′-CGTGTCTCCTCCTCCCATT-3′ and 5′-CCGCTGTTATCAGCACCAG-3′ for a CSL-binding site at the *HES1* promoter^88^, 5′-TGGATCCAATCCTATTGCCC-3′ and 5′-CGCAGCAGTTGGAAGTGTTT-3′ for an off target control for *HES1* ^88^. Data represent three independent experiments.

### Statistical analyses

Data from experiments were presented as mean ± standard error (*n* = 3–6) in real-time RT-PCR, luciferase assays, flow cytometry and IHC labeling index or mean ± standard deviation (*n* = 8–12) in xenograft transplantation tumor analyses. GraphPad Prism 7 (GraphPad Software, La Jolla, CA) or Stata Version 14 (StataCorp, College Station, TX) software were used for statistical analyses. The two-tailed Student’s *t-*test was used for paired comparisons. Fisher’s exact test was used for percentage comparisons in xenograft experiments. ANOVA with Tukey’s post hoc test was used for multiple pairwise comparisons. *P* < 0.05 was considered significant. The Kappa Statistic was used to evaluate concordance between NOTCH1-positive IHC staining as classified by independent antibodies. Survival curves were estimated using the Kaplan–Meier method and plotted. Candidate predictor variables were tested at *P* < 0.05 by univariate and multivariate Cox Regression using the *z*-score corresponding to the hazard ratio.

### Data availability

Microarray data were deposited at the NCBI Gene Expression Omnibus (http://www.ncbi.nlm.nih.gov/geo) under accession number GSE37994. All data sets are available from the authors upon request.

## Electronic supplementary material

Supplementary Information

Peer Review File

Description of Additional Supplementary Files

Supplementary Data 1

## References

[1] Kopan R, Ilagan MX (2009). The canonical Notch signaling pathway: unfolding the activation mechanism. Cell.

[2] Blanpain C, Lowry WE, Pasolli HA, Fuchs E (2006). Canonical notch signaling functions as a commitment switch in the epidermal lineage. Genes Dev..

[3] Rangarajan A (2001). Notch signaling is a direct determinant of keratinocyte growth arrest and entry into differentiation. EMBO J..

[4] Ohashi S (2010). NOTCH1 and NOTCH3 coordinate esophageal squamous differentiation through a CSL-dependent transcriptional network. Gastroenterology.

[5] Demehri S, Turkoz A, Kopan R (2009). Epidermal Notch1 loss promotes skin tumorigenesis by impacting the stromal microenvironment. Cancer Cell.

[6] Agrawal N (2011). Exome sequencing of head and neck squamous cell carcinoma reveals inactivating mutations in NOTCH1. Science.

[7] Stransky N (2011). The mutational landscape of head and neck squamous cell carcinoma. Science.

[8] Dotto GP, Rustgi AK (2016). Squamous cell cancers: a unified perspective on biology and genetics. Cancer Cell.

[9] Nicolas M (2003). Notch1 functions as a tumor suppressor in mouse skin. Nat. Genet..

[10] Zhong R (2015). Notch1 activation or loss promotes HPV-induced oral tumorigenesis. Cancer Res..

[11] Ohashi S (2011). A NOTCH3-mediated squamous cell differentiation program limits expansion of EMT-competent cells that express the ZEB transcription factors. Cancer Res..

[12] Kagawa S (2015). Cellular senescence checkpoint function determines differential Notch1-dependent oncogenic and tumor-suppressor activities. Oncogene.

[13] Yuan X (2015). Notch signaling: an emerging therapeutic target for cancer treatment. Cancer Lett..

[14] Ronchini C, Capobianco AJ (2001). Induction of cyclin D1 transcription and CDK2 activity by Notch(ic): implication for cell cycle disruption in transformation by Notch(ic). Mol. Cell Biol..

[15] Zavadil J, Cermak L, Soto-Nieves N, Bottinger EP (2004). Integration of TGF-beta/Smad and Jagged1/Notch signalling in epithelial-to-mesenchymal transition. EMBO J..

[16] Niessen K (2008). Slug is a direct Notch target required for initiation of cardiac cushion cellularization. J. Cell Biol..

[17] Sahlgren C, Gustafsson MV, Jin S, Poellinger L, Lendahl U (2008). Notch signaling mediates hypoxia-induced tumor cell migration and invasion. Proc. Natl. Acad. Sci. USA.

[18] Ansieau S (2008). Induction of EMT by twist proteins as a collateral effect of tumor-promoting inactivation of premature senescence. Cancer Cell.

[19] Ohashi S (2010). Epidermal growth factor receptor and mutant p53 expand an esophageal cellular subpopulation capable of epithelial-to-mesenchymal transition through ZEB transcription factors. Cancer Res..

[20] Ohashi S (2015). Recent advances from basic and clinical studies of esophageal squamous cell carcinoma. Gastroenterology.

[21] Basu D (2010). Evidence for mesenchymal-like sub-populations within squamous cell carcinomas possessing chemoresistance and phenotypic plasticity. Oncogene.

[22] Uchikado Y (2005). Slug expression in the E-cadherin preserved tumors is related to prognosis in patients with esophageal squamous cell carcinoma. Clin. Cancer Res..

[23] Usami Y (2008). Snail-associated epithelial-mesenchymal transition promotes oesophageal squamous cell carcinoma motility and progression. J. Pathol..

[24] Mani SA (2008). The epithelial-mesenchymal transition generates cells with properties of stem cells. Cell.

[25] Celia-Terrassa T (2012). Epithelial-mesenchymal transition can suppress major attributes of human epithelial tumor-initiating cells. J. Clin. Invest..

[26] Al-Hajj M, Wicha MS, Benito-Hernandez A, Morrison SJ, Clarke MF (2003). Prospective identification of tumorigenic breast cancer cells. Proc. Natl. Acad. Sci. USA.

[27] Zhao JS (2011). Tumor initiating cells in esophageal squamous cell carcinomas express high levels of CD44. PLoS ONE.

[28] Biddle A (2011). Cancer stem cells in squamous cell carcinoma switch between two distinct phenotypes that are preferentially migratory or proliferative. Cancer Res..

[29] Prince ME (2007). Identification of a subpopulation of cells with cancer stem cell properties in head and neck squamous cell carcinoma. Proc. Natl. Acad. Sci. USA.

[30] Whelan KA (2017). Autophagy supports generation of cells with high CD44 expression via modulation of oxidative stress and Parkin-mediated mitochondrial clearance. Oncogene.

[31] Kinugasa H (2015). Mitochondrial SOD2 regulates epithelial-mesenchymal transition and cell populations defined by differential CD44 expression. Oncogene.

[32] Zavadil J, Bottinger EP (2005). TGF-beta and epithelial-to-mesenchymal transitions. Oncogene.

[33] Brabletz S (2011). The ZEB1/miR-200 feedback loop controls Notch signalling in cancer cells. EMBO J..

[34] Postigo AA (2003). Opposing functions of ZEB proteins in the regulation of the TGFbeta/BMP signaling pathway. EMBO J..

[35] Postigo AA, Depp JL, Taylor JJ, Kroll KL (2003). Regulation of Smad signaling through a differential recruitment of coactivators and corepressors by ZEB proteins. EMBO J..

[36] Vallejo DM, Caparros E, Dominguez M (2011). Targeting Notch signalling by the conserved miR-8/200 microRNA family in development and cancer cells. EMBO J..

[37] Zhang T (2016). A genetic cell context-dependent role for ZEB1 in lung cancer. Nat. Commun..

[38] Wang T (2014). Notch-1-mediated esophageal carcinoma EC-9706 cell invasion and metastasis by inducing epithelial-mesenchymal transition through Snail. Tumour Biol..

[39] Tang XH, Scognamiglio T, Gudas LJ (2013). Basal stem cells contribute to squamous cell carcinomas in the oral cavity. Carcinogenesis.

[40] Chang CJ (2011). p53 regulates epithelial-mesenchymal transition and stem cell properties through modulating miRNAs. Nat. Cell Biol..

[41] Lefort K (2007). Notch1 is a p53 target gene involved in human keratinocyte tumor suppression through negative regulation of ROCK1/2 and MRCKalpha kinases. Genes Dev..

[42] Maltzman W, Czyzyk L (1984). UV irradiation stimulates levels of p53 cellular tumor antigen in nontransformed mouse cells. Mol. Cell Biol..

[43] Yugawa T (2007). Regulation of Notch1 gene expression by p53 in epithelial cells. Mol. Cell Biol..

[44] Maillard I (2004). Mastermind critically regulates Notch-mediated lymphoid cell fate decisions. Blood.

[45] Blokzijl A (2003). Cross-talk between the Notch and TGF-beta signaling pathways mediated by interaction of the Notch intracellular domain with Smad3. J. Cell Biol..

[46] Nam JS (2008). An anti-transforming growth factor beta antibody suppresses metastasis via cooperative effects on multiple cell compartments. Cancer Res..

[47] Ovcharenko I, Nobrega MA, Loots GG, Stubbs L (2004). ECR Browser: a tool for visualizing and accessing data from comparisons of multiple vertebrate genomes. Nucleic Acids Res..

[48] Cui H, Kong Y, Xu M, Zhang H (2013). Notch3 functions as a tumor suppressor by controlling cellular senescence. Cancer Res..

[49] Bao B (2011). Notch-1 induces epithelial-mesenchymal transition consistent with cancer stem cell phenotype in pancreatic cancer cells. Cancer Lett..

[50] Wellner U (2009). The EMT-activator ZEB1 promotes tumorigenicity by repressing stemness-inhibiting microRNAs. Nat. Cell Biol..

[51] Rhim AD (2012). EMT and dissemination precede pancreatic tumor formation. Cell.

[52] Pickup M, Novitskiy S, Moses HL (2013). The roles of TGFβ in the tumour microenvironment. Nat. Rev. Cancer.

[53] Osipo C, Golde TE, Osborne BA, Miele LA (2008). Off the beaten pathway: the complex cross talk between Notch and NF-kappaB. Lab. Invest..

[54] Gu F (2010). Expression of Stat3 and Notch1 is associated with cisplatin resistance in head and neck squamous cell carcinoma. Oncol. Rep..

[55] Hijioka H (2010). Upregulation of Notch pathway molecules in oral squamous cell carcinoma. Int. J. Oncol..

[56] Zagouras P, Stifani S, Blaumueller CM, Carcangiu ML, Artavanis-Tsakonas S (1995). Alterations in Notch signaling in neoplastic lesions of the human cervix. Proc. Natl. Acad. Sci. USA.

[57] Chaffer CL (2013). Poised chromatin at the ZEB1 promoter enables breast cancer cell plasticity and enhances tumorigenicity. Cell.

[58] Zhang J (2014). TGF-beta-induced epithelial-to-mesenchymal transition proceeds through stepwise activation of multiple feedback loops. Sci. Signal..

[59] Lu M, Jolly MK, Levine H, Onuchic JN, Ben-Jacob E (2013). MicroRNA-based regulation of epithelial-hybrid-mesenchymal fate determination. Proc. Natl. Acad. Sci. USA.

[60] Naganuma S (2012). Notch receptor inhibition reveals the importance of cyclin D1 and Wnt signaling in invasive esophageal squamous cell carcinoma. Am. J. Cancer Res..

[61] Alcolea MP (2014). Differentiation imbalance in single oesophageal progenitor cells causes clonal immortalization and field change. Nat. Cell Biol..

[62] Li K (2008). Modulation of Notch signaling by antibodies specific for the extracellular negative regulatory region of NOTCH3. J. Biol. Chem..

[63] Wu Y (2010). Therapeutic antibody targeting of individual Notch receptors. Nature.

[64] Teicher BA (2007). Transforming growth factor-beta and the immune response to malignant disease. Clin. Cancer Res..

[65] Whelan KA (2016). Autophagy mediates epithelial cytoprotection in eosinophilic oesophagitis. Gut.

[66] Basu D (2013). EGFR inhibition promotes an aggressive invasion pattern mediated by mesenchymal-like tumor cells within squamous cell carcinomas. Mol. Cancer Ther..

[67] Lee JJ (2010). Hypoxia activates the cyclooxygenase-2-prostaglandin E synthase axis. Carcinogenesis.

[68] Liu K (2013). Sox2 cooperates with inflammation-mediated Stat3 activation in the malignant transformation of foregut basal progenitor cells. Cell Stem Cell.

[69] Yang Y, Katz JP (2016). *KL*F4 is downregulated but not mutated during human esophageal squamous cell carcinogenesis and has tumor stage-specific functions. Cancer Biol. Ther..

[70] Griffin MC, Robinson RA, Trask DK (2003). Validation of tissue microarrays using p53 immunohistochemical studies of squamous cell carcinoma of the larynx. Mod. Pathol..

[71] Stairs DB (2011). Deletion of p120-catenin results in a tumor microenvironment with inflammation and cancer that establishes it as a tumor suppressor gene. Cancer Cell.

[72] Olive KP (2004). Mutant p53 gain of function in two mouse models of Li-Fraumeni syndrome. Cell.

[73] Tang XH, Knudsen B, Bemis D, Tickoo S, Gudas LJ (2004). Oral cavity and esophageal carcinogenesis modeled in carcinogen-treated mice. Clin. Cancer Res..

[74] Kalabis J (2012). Isolation and characterization of mouse and human esophageal epithelial cells in 3D organotypic culture. Nat. Protoc..

[75] Facompre ND (2016). JARID1B enables transit between distinct states of the stem-like cell population in oral cancers. Cancer Res..

[76] Jeffries S, Capobianco AJ (2000). Neoplastic transformation by Notch requires nuclear localization. Mol. Cell Biol..

[77] Sanjana NE, Shalem O, Zhang F (2014). Improved vectors and genome-wide libraries for CRISPR screening. Nat. Methods.

[78] Takaoka M (2004). Ha-Ras(G12V) induces senescence in primary and immortalized human esophageal keratinocytes with p53 dysfunction. Oncogene.

[79] Kim S-H (2006). Tumorigenic conversion of primary human esophageal epithelial cells using oncogene combinations in the absence of exogenous Ras. Cancer Res..

[80] Natsuizaka M (2010). Insulin-like growth factor-binding protein-3 promotes transforming growth factor-{beta}1-mediated epithelial-to-mesenchymal transition and motility in transformed human esophageal cells. Carcinogenesis.

[81] Nishihira T, Hashimoto Y, Katayama M, Mori S, Kuroki T (1993). Molecular and cellular features of esophageal cancer cells. J. Cancer Res. Clin. Oncol..

[82] Sashiyama H (2001). Immortalization of human esophageal keratinocytes by E6 and E7 of human papillomavirus type 16. Int. J. Oncol..

[83] Harada H (2003). Telomerase induces immortalization of human esophageal keratinocytes without p16INK4a inactivation. Mol. Cancer Res..

[84] DeWard AD, Cramer J, Lagasse E (2014). Cellular heterogeneity in the mouse esophagus implicates the presence of a nonquiescent epithelial stem cell population. Cell Rep..

[85] Tanaka K (2016). ALDH2 modulates autophagy flux to regulate acetaldehyde-mediated toxicity thresholds. Am. J. Cancer Res..

[86] Natsuizaka M (2014). IGFBP3 promotes esophageal cancer growth by suppressing oxidative stress in hypoxic tumor microenvironment. Am. J. Cancer Res..

[87] Drake JM (2010). ZEB1 coordinately regulates laminin-332 and {beta}4 integrin expression altering the invasive phenotype of prostate cancer cells. J. Biol. Chem..

[88] Yashiro-Ohtani Y (2009). *Pre-TCR signaling inac*tivates Notch1 transcription by antagonizing E2A. Genes Dev..

