# Supplementary material for: Interplay between Notch1 and Notch3 promotes EMT and tumor initiation in squamous cell carcinoma
Source: Nat Commun. 2017 Nov 24;8:1758. doi: 10.1038/s41467-017-01500-9 (PMC5700926; doi:10.1038/s41467-017-01500-9)
Supplement: Supplementary file 1 — Supplementary Information [file 41467_2017_1500_MOESM1_ESM.pdf]

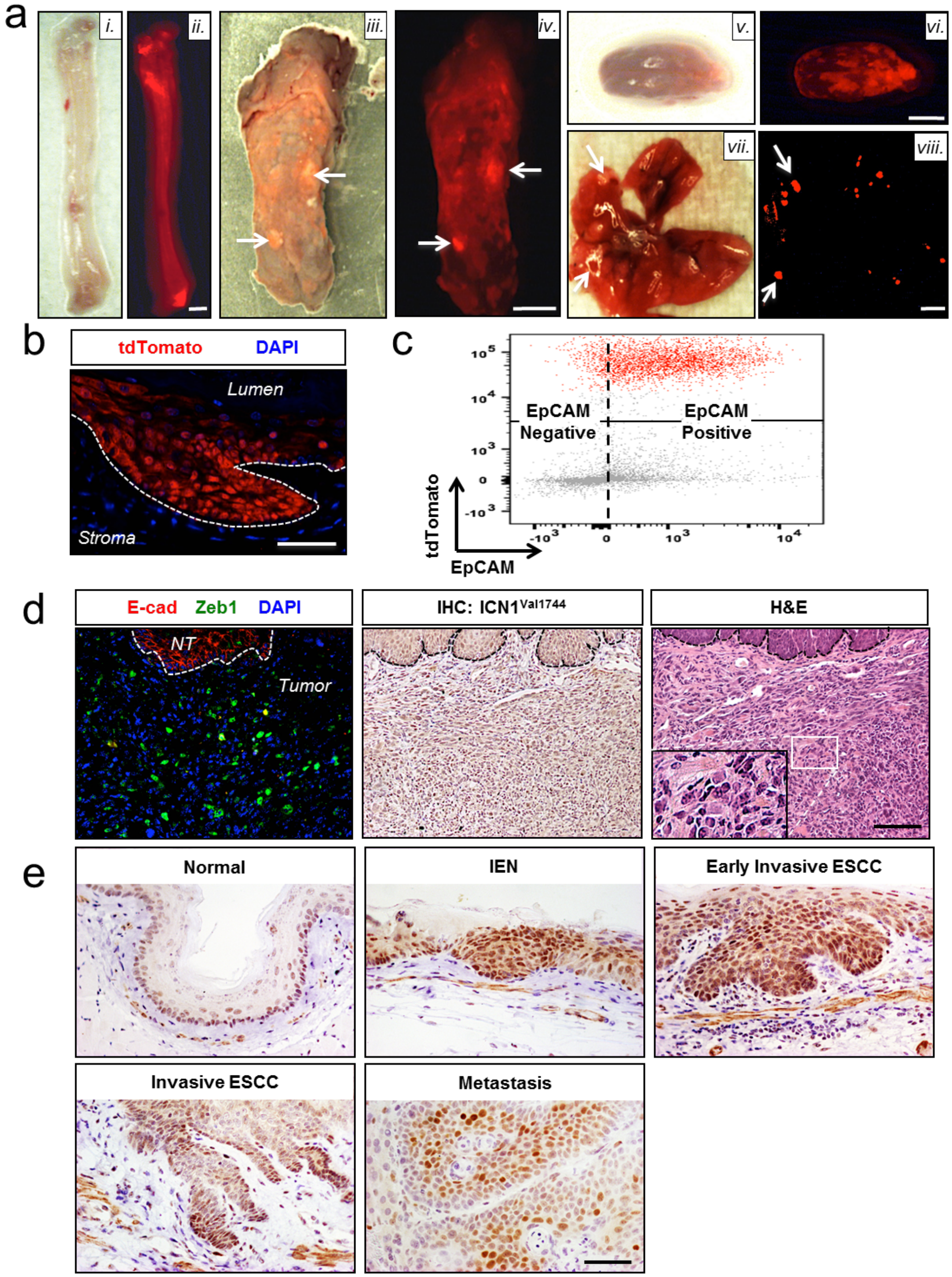

Supplementary Figure 1 Analyses of EMT and ICN1 expression in 4NQO-induced lesions labeled with tdTomato (**a**) Macroscopic and fluorescent images of tdTomato-labeled normal esophageal mucosa (i and ii) and 4NQO-induced primary and metastatic lesions in the tongue (iii and iv), the lymph node (v and vi) and the liver (vii and viii). Arrows indicate tumors. Scale bars, 1 mm. (**b**) Representative IF image documenting tdTomato expression in 4NQO-induced early neoplastic lesion. Scale bar, 50 μm. (**c**) Flow cytometry scatter plot for tdTomato and EpCAM expression in cells from a representative 4NQO-induced tumor. 6.8 ± 1.2% $tdTomato^{Pos}EpCAM^{Neg}$ (upper left quadrant); 80.4 ± 5.9% $tdTomato^{Pos}EpCAM^{Pos}$ (upper right quadrant) represents average distribution in live cells across three independent tumors. (**d**) Representative multicolor IF image for Zeb1 and E-cadherin (E-cad), IHC for ICN1 ($ICN1^{Val1744}$) and H&E staining of highly invasive ESCC cells (magnified in the inset) from esophagus-targeted *$p53^{R172H/wt}$* mice (n=4). Note that E-cadherin (E-cad) is expressed in the non-tumor (NT) superficial epithelium, but not in tumors. Scale bar, 50 μm. (**e**) Representative ICN1 ($ICN1^{Val1744}$) IHC images for indicated 4NQO-induced lesions. IEN, intraepithelial neoplasia (dysplasia). Scale bar, 50 μm. Dashed lines in **b** and **d** denote interface between invasive ESCC cells and surrounding stroma.

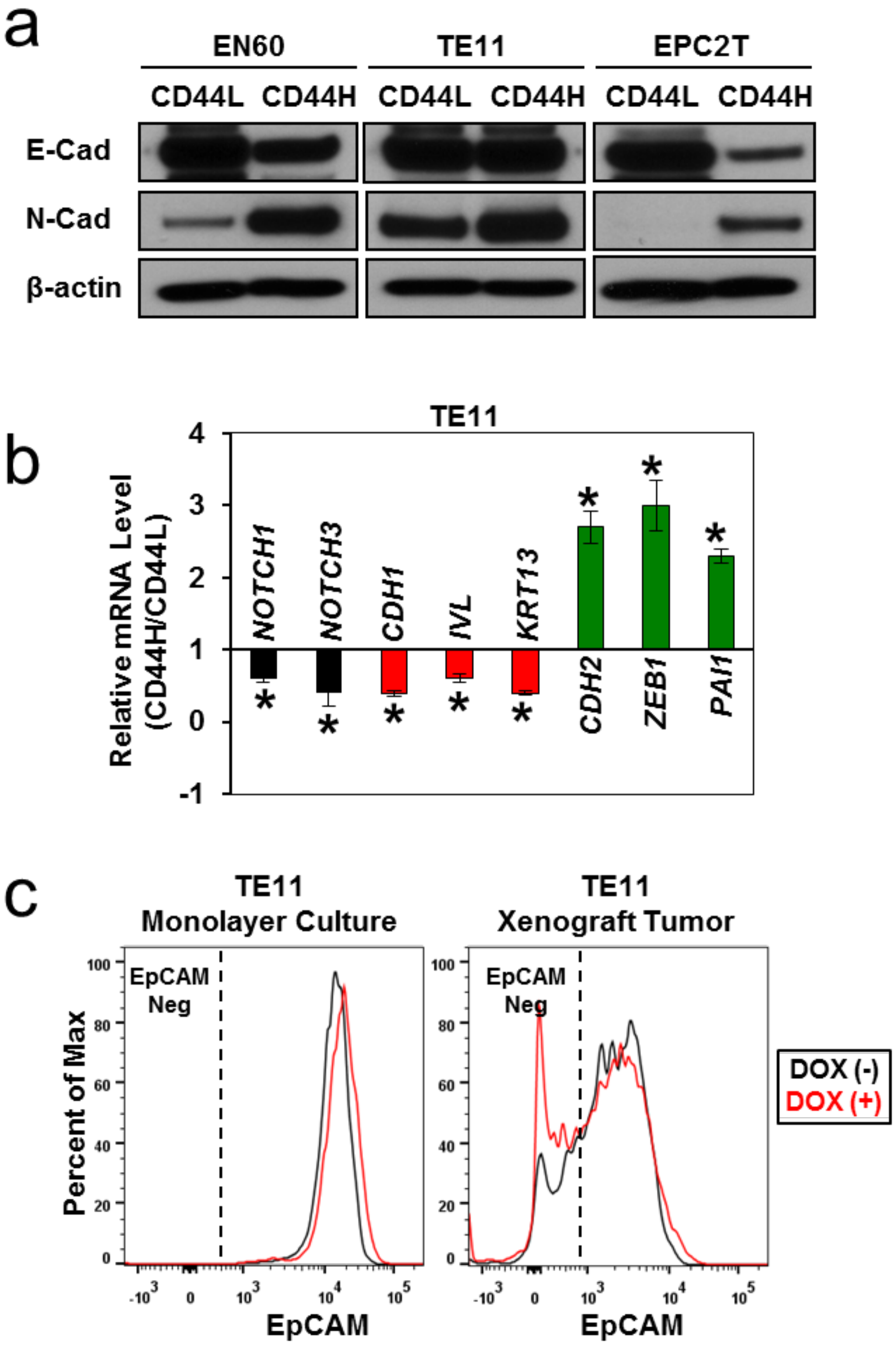

Supplementary Figure 2 Notch-dependent tumor growth and mesenchymal characteristics of TE11 cells (**a**) Immunoblot analysis of FACS-purified CD44L and CD44H cells from indicated cell lines. β-actin serves a loading control in immunoblots. E-cad, E-cadherin; N-cad, N-cadherin. (**b**) qRT-PCR analysis for indicated genes comparing FACS-purified CD44L and CD44H cells from a representative TE11 xenograft tumor. CD44L cell mRNA level for each gene was set to 1. *, $P<0.01$ vs. CD44L, n=3. (**f**) Representative flow cytometry histogram plots for EpCAM expression in TE11 cells with or without DOX-mediated expression of ectopic ICN1 in monolayer culture and xenograft tumors. tRFP-expressing TE11 tumors were analyzed to exclude host-derived stromal cells. (**g**) Flow cytometry analysis for EpCAM-negative cells in TE11 with *NOTCH1* deletion ($N1^{\Delta}$) or wild-type control ($N1^{WT}$). Cells were treated with or without 5 ng/ml TGFβ for 72 h. *, $P<0.05$ vs. TGFβ (-); #, $P<0.0001$ vs. $N1^{WT}$ and TGFβ (+), n=3. Data is presented as mean ± s.e.m. in **a** and **b**; and mean ± s.d. in **e** and **g**. ANOVA with Tukey's post-hoc test was used for multiple comparisons in **a**, **b and g**. Fisher's exact test was used for percentage comparisons in **c**. Student's t-test was used for paired data comparisons in **e**.

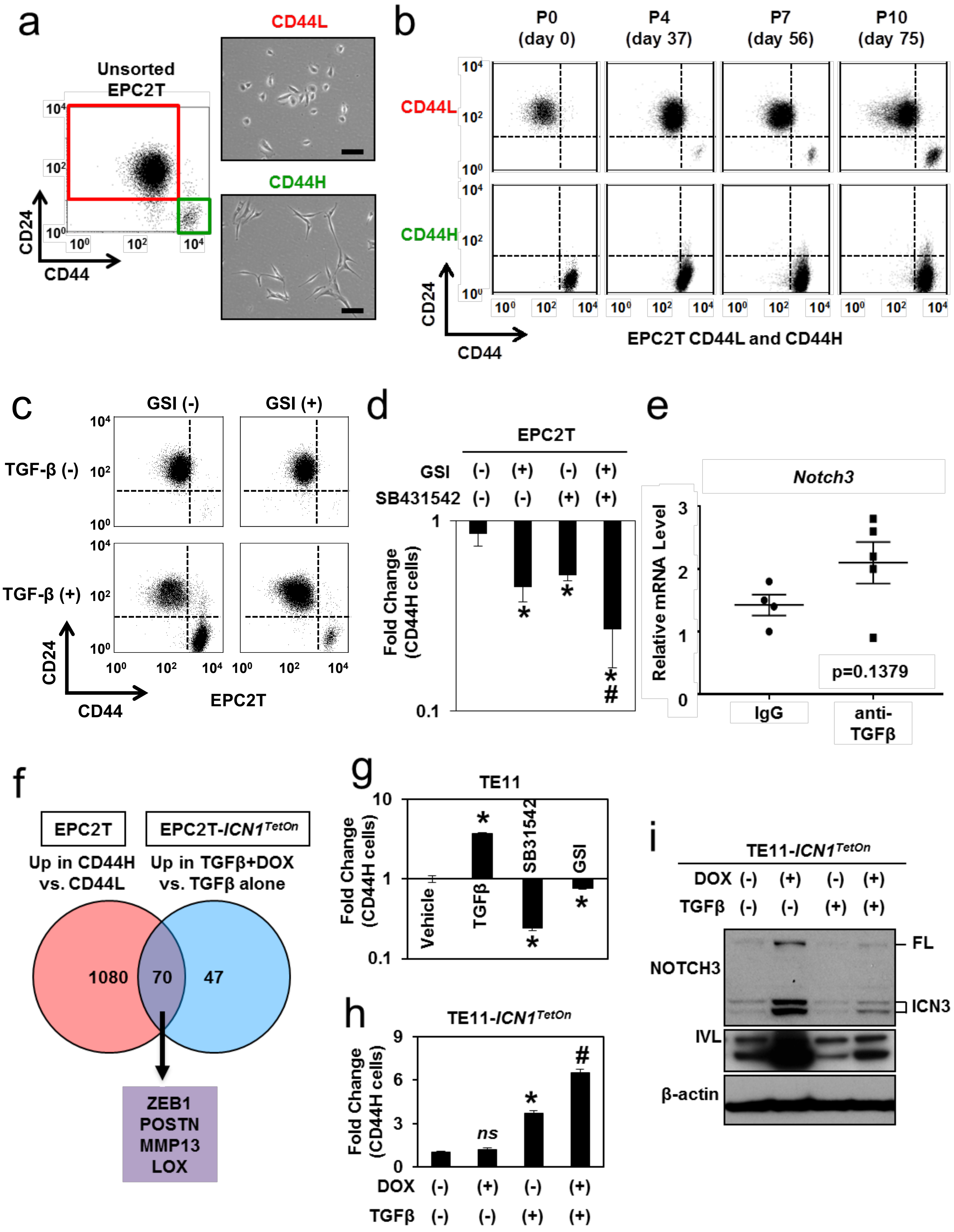
a
Unsorted EPC2T
CD44L
CD44H
CD24
CD44
b
P0 (day 0)
P4 (day 37)
P7 (day 56)
P10 (day 75)
CD44L
CD44H
CD24
CD44
EPC2T CD44L and CD44H
c
GSI (-)
GSI (+)
TGF-β (-)
TGF-β (+)
CD24
CD44
EPC2T
d
EPC2T
GSI (-) (+) (-) (+)
SB431542 (-) (-) (+) (+)
Fold Change (CD44H cells)
e
Notch3
Relative mRNA Level
p=0.1379
IgG
anti-TGFβ
f
EPC2T
EPC2T-ICN1TetOn
Up in CD44H vs. CD44L
Up in TGFβ+DOX vs. TGFβ alone
1080
70
47
ZEB1
POSTN
MMP13
LOX
g
TE11
Fold Change (CD44H cells)
Vehicle
TGFβ
SB31542
GSI
h
TE11-ICN1TetOn
Fold Change (CD44H cells)
ns
DOX (-) (+) (-) (+)
TGFβ (-) (-) (+) (+)
i
TE11-ICN1TetOn
DOX (-) (+) (-) (+)
TGFβ (-) (-) (+) (+)
NOTCH3
FL
ICN3
IVL
β-actin

Supplementary Figure 3 Analysis of CD44L and CD44H populations in EPC2T and EN60 cells

(**a**) and (**b**) Representative flow cytometry scatter plots of EPC2T cells with phase-contrast photomicrographs of CD44L and CD44H cells taken following FACS purification. Each subpopulation was passaged as noted with time periods indicated. (**c**) Representative flow cytometry scatter plots of purified CD44L cells treated with TGFβ along with or without GSI for 7 days. (**d**) Bar graph showing frequency of CD44H induction from FACS-purified EPC2T CD44L cells treated with SB431542 along with or without GSI for 7 days. *, $P<0.05$ vs. SB431542 (-) and GSI (-); #, $P<0.05$ vs. SB431542 (+) and GSI (-); n=3. (**e**) $p53^{+/+}$ mice were treated with 4NQO for 16 weeks. Six weeks following 4NQO withdrawal, mice were treated with ID11 anti-TGFβ blocking antibody or control IgG via intraperitoneal injection 3 times each week for a period of 2 weeks. Esophageal epithelium was peeled from a centrally-located ~1-2cm section of tissue and analyzed for expression of *Notch3* by qRT-PCR. (**f**) Venn diagram showing 1080 genes exhibiting upregulation specifically in CD44H cells as compared to CD44L cells in EPC2T and 47 genes exhibiting upregulation specifically in EPC2T cells treated with TGFβ and DOX in combination as compared to those treated with TGFβ alone. 70 genes, including *ZEB1*, Periostin (*POSTN*), Lysyl Oxidase (*LOX*) and Matrix metalloproteinase (*MMP*)-13, are commonly upregulated under these two conditions. (**g-i**) Analysis of parental TE11 and derivative with DOX-inducible ICN1 following indicated treatment conditions for 7 days. Bar graphs in **g** and **h** show the frequency of CD44H cell induction determined by flow cytometry. *, $P<0.05$ vs. vehicle in **g**, n=3. In **h**, *, $P<0.05$ vs. TGFβ (-) and DOX (-); #, $P<0.05$ vs. TGFβ (-) and DOX (+); *ns*, not significant vs. TGFβ (-) and DOX (-); n=3. In **i,** Immunoblot analysis determined level of indicated proteins. β-actin served as a loading control. FL, full-length NOTCH3; bracket indicates doublet (ICN3) that appeared consistently and may represent a posttranslational modification. All bar diagrams indicate mean ± s.d. Dot plot in **e** depicts values for each independent animal with mean ± s.e.m shown. ANOVA with Tukey's post-hoc test was used for multiple comparisons in **d**, **g** and **h**. Student's t-test was used for paired data comparisons in **e**.

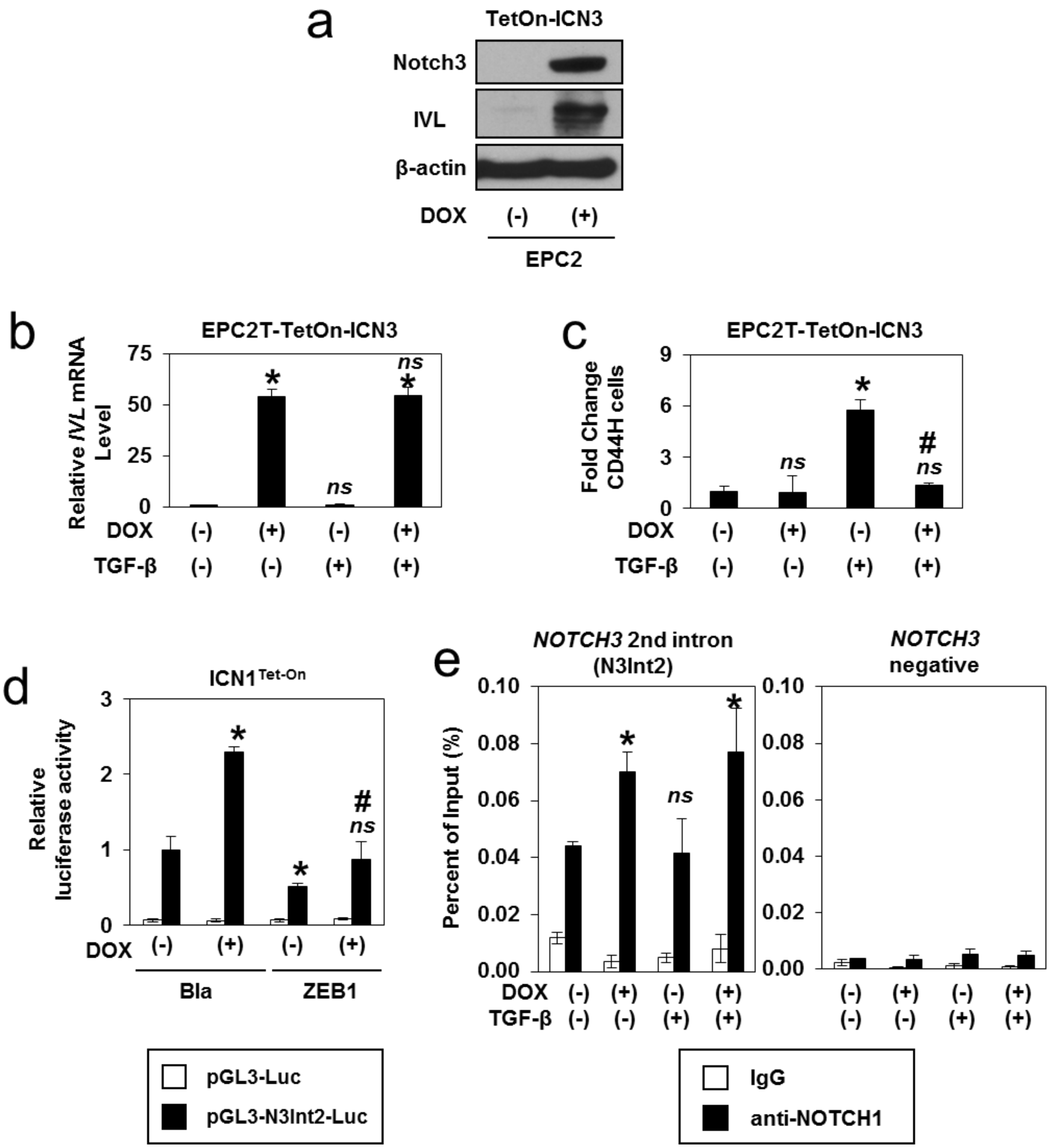

Supplementary Figure 4 Functional consequences of ectopic ICN3 expression and the effect of ICN1 expression upon *NOTCH3* transcriptional regulation. (**a-c**) DOX-induced ectopic ICN3 expression promotes IVL expression in EPC2T cells and prevents TGFβ-mediated CD44H cell induction. In **a,** immunoblot analysis confirms ICN3 and concurrent IVL induction with β-actin as a loading control. In **b,** qRT-PCR analysis for *IVL* mRNA expression in EPC2T cells cultured under indicated conditions. mRNA level for DOX (-) and TGFβ (-) was set to 1. *, P<0.05 vs. TGFβ (-) and DOX (-); *ns*, not significant vs. TGFβ (-) and DOX (+); n=3. In **c,** bar graph demonstrates frequency of CD44H cell induction in EPC2T cells under indicated conditions. *, P<0.05 vs. TGFβ (-) and DOX (-); *ns*, not significant vs. TGFβ (-) and DOX (-); #, P<0.05 vs. TGFβ (+) and DOX (-); n=3. Treatments with TGFβ and DOX were done for 72h in **a,** and 7 days in **b** and **c**. (**d**) Transfection assays for *pGL3-N3Int2-luc* luciferase reporter activity in EPC2T cells with DOX-inducible ICN1 along with or without ectopic ZEB1 expression. *bla*, empty vector control for ZEB1. *, P<0.05 vs. *pGL3-N3Int2-luc* and *bla* and DOX (-), n=3. #, P<0.05 vs. *pGL3-N3Int2-luc* and *bla* and DOX (+); *ns*, not significant vs. *pGL3-N3Int2-luc* and ZEB1 and DOX (-); n=4. (**e**) ChIP assays for ICN1 binding to the *N3Int2* region in EPC2T cells with DOX-inducible ICN1. *, P<0.05 vs. anti-NOTCH1, TGFβ (-) and DOX (-); *ns*, not significant 1 vs. anti-NOTCH1, TGFβ (-) and DOX (-); n=3. Cells were stimulated with or without DOX or TGFβ for 72 h prior to luciferase assays in **d** and ChIP assays in **e**. All bar diagrams represent mean ± s.d. ANOVA with Tukey's post-hoc test was used for multiple comparisons in **b-e**.

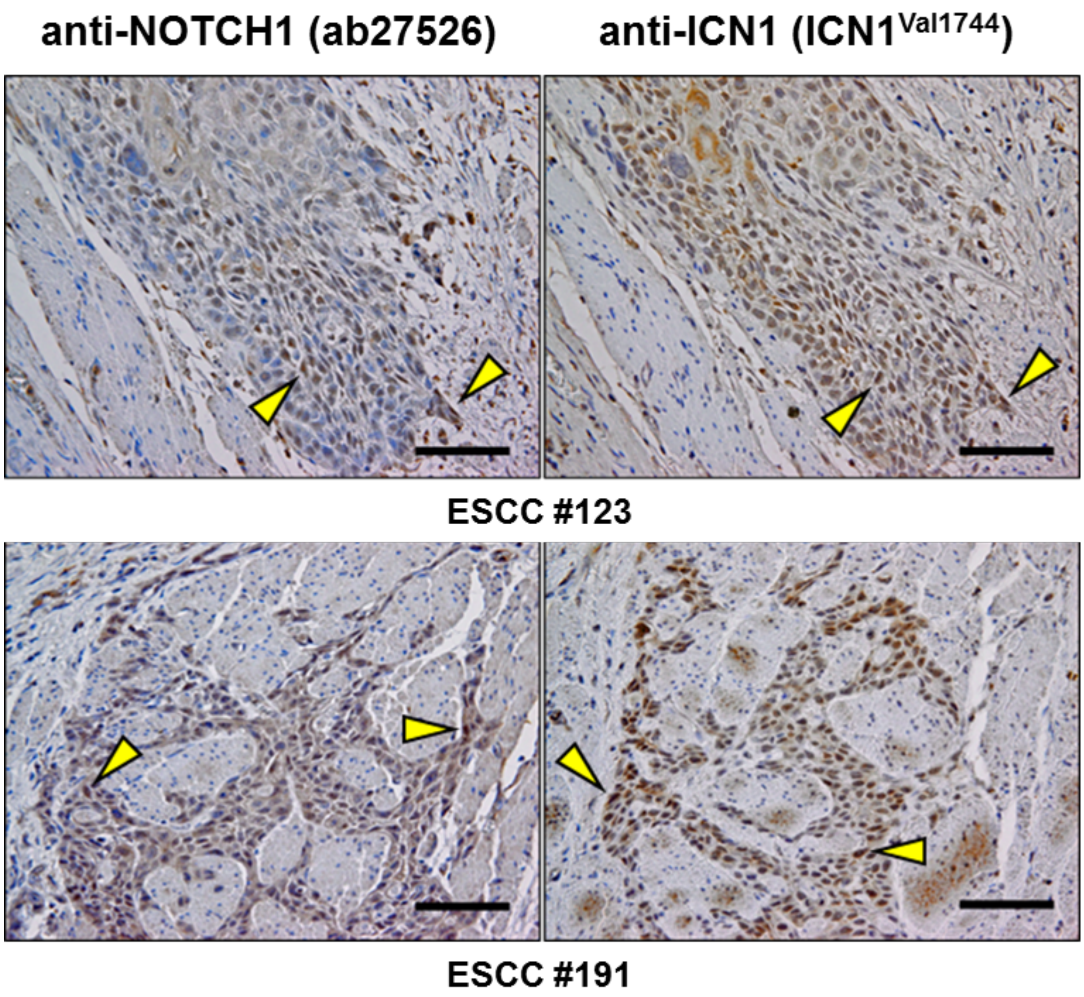

Supplementary Figure 5 NOTCH1 antibody comparison IHC images of NOTCH1 staining for two representative ESCC cases (top and bottom). Two independent antibodies (anti-ICN1^{Val1744}) and (anti-full length NOTCH1, ab27562) showed a high concordance rate with regard to nuclear and cytoplasmic NOTCH1 staining in invasive ESCC. ESCC cases are listed in in **Supplementary Data 1**.

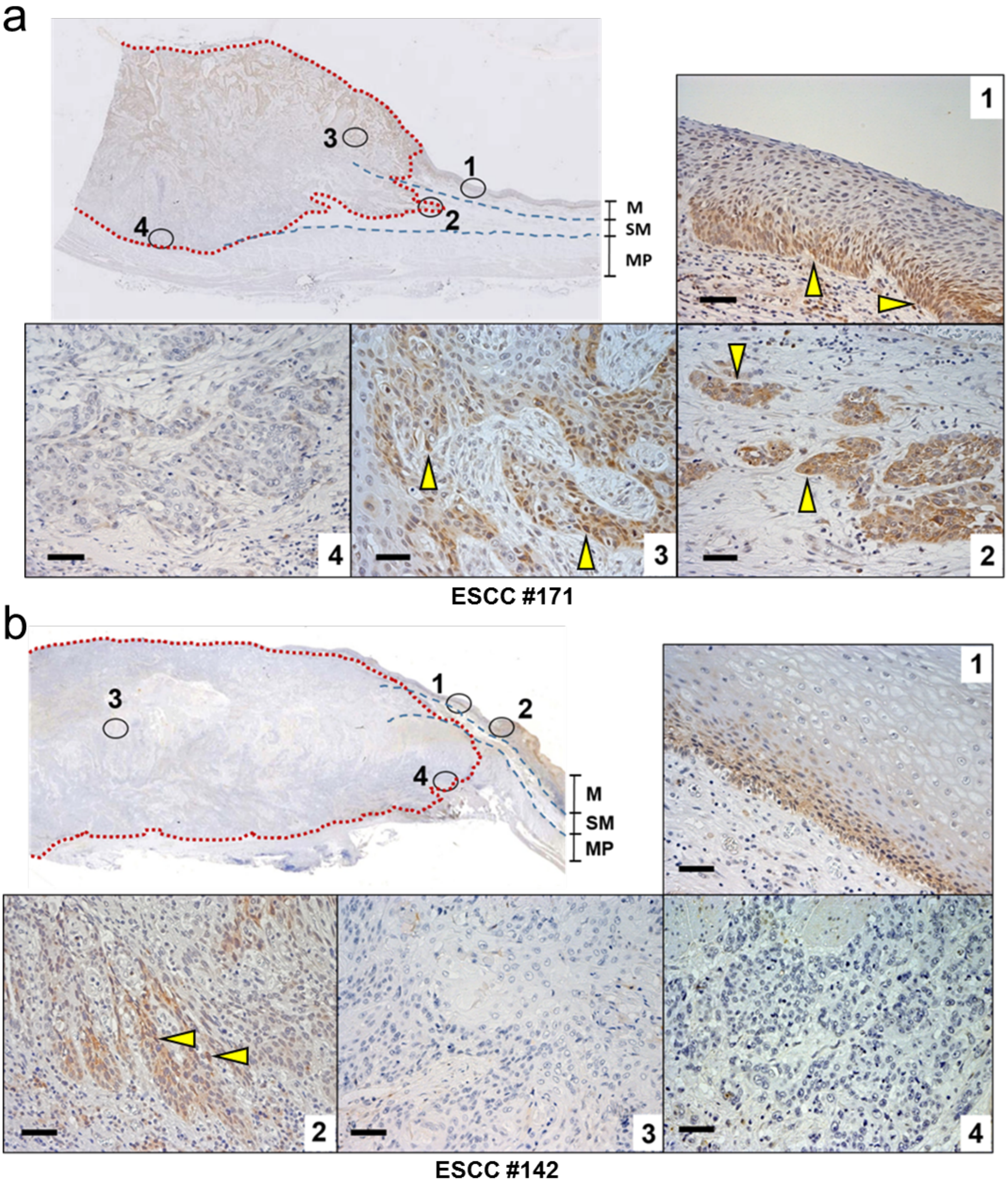

Supplementary Figure 6 IHC analysis of human ESCC samples reveals heterogeneous ICN1 expression Low and high magnifications of representative IHC slides classified as ICN1 (+) and ICN1 (-), respectively. In (**a**), ICN1-positivity is apparent in the superficial dysplastic lesion (area 1), the deep invasive tumor front (area 2), and in intratumoral invasive ESCC cells (area 3). The main tumor also contained ICN1-negative ESCC cells (area 4), revealing heterogeneous ICN1 expression in ESCC. In (**b**), ICN1-negative ESCC cells are present in the main tumor (area 3) and at the invasive tumor front (area 4). ICN1-positivity was restricted to superficial micro-invasive carcinoma (area 2) and adjacent normal mucosa (area 1). A nonsense mutation in NOTCH1 was confirmed in this tumor. ESCC cases in **a**, **b** are listed in **Supplementary Data 1.**

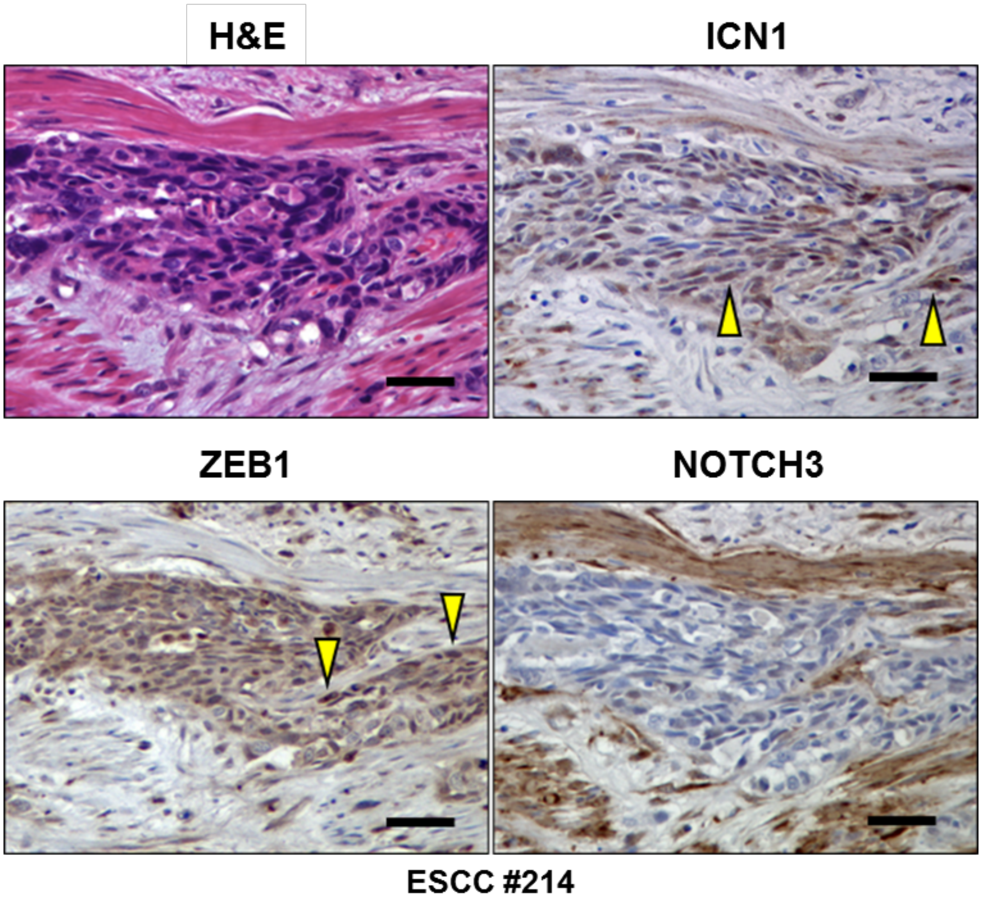

Supplementary Figure 7 IHC analysis of human ESCC samples reveals expression of ICN1 and ZEB1 concurrent with NOTCH3 downregulation in invasive tumor nests Representative IHC images for ICN1, ZEB1 and NOTCH3 staining in serial sections from an ESCC case showing ICN1-positive ESCC cells in deep invasive tumor nests. NOTCH3 was positive in stromal smooth muscle surrounding the ESCC cell nest. Scale bars, 50μm. ESCC cases are listed in **Supplementary Data 1**.

.

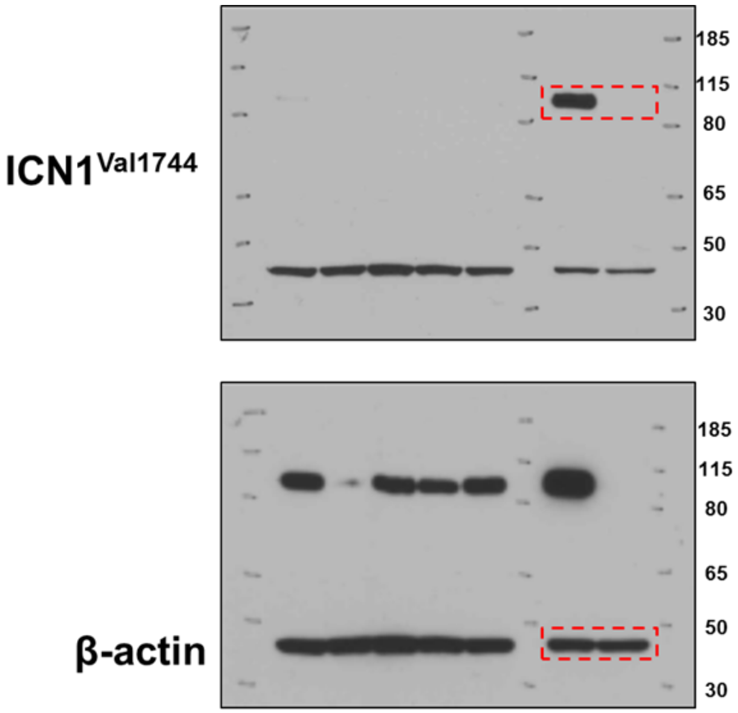

Supplementary Figure 8 Uncropped scans of blots from primary figures Uncropped scans of immunoblots for the activated form of Notch1 (ICN1Val[1744]) and the loading control β-Actin used in Figure 2a. Hatched boxes denote the area that is shown in the original figure.

Supplementary Table 1

Univariate and multivariate analysis of clinicopathologic factors affecting overall survival rate

Variables	N	5-years survival	Univariate analysis	Multivariate analysis	
		Rate (%)	*p*-value	HR (CI 95%)	*p*-value
**Tumor depth**					
pT1,2	68	51.09	**＜0.0001**	2.449	**＜0.0001**
pT3,4	117	20.93		(1.587 - 3.781)	
**Lymph node metastasis**					
Negative	73	50.53	**＜0.0001**	1.425	0.202
Positive	112	19.34		(0.826 - 2.459)	
**Lymphatic invasion**					
Negative	58	44.95	**＜0.001**	1.186	0.523
Positive	127	26.42		(0.702 - 2.004)	
Venous invasion					
Negative	100	35.14	0.245	0.935	0.732
Positive	85	28.92		(0.634 - 1.377)	
**Distant metastasis**					
Negative	139	39.39	**＜0.0001**	1.676	**0.027**
Positive	46	11.36		(1.062 - 2.645)	
**ICN1 expression**					
Negative	121	39.69	**<0.0001**	2.027	**0.001**
Positive	64	17.64		(1.350 - 3.045)	

N, number of patient; CI, confidence interval.

Analysis of 185 therapy naïve ESCC patients (case #1-#185) in **Supplementary Data 1.**

Supplementary Table 2
Relationship between ICN1 expression and clinicopathologic findings

		**ICN1 expression**				
		**Negative(n=126)**		**Positive (n=64)**		
**Factors**	**Total**	**No.**	**%**	**No.**	**%**	***p***
Gender						
Male	169	107	63.3	62	36.7	0.0520
Female	16	14	87.5	2	12.5	
Tumor Location						
Upper	34	17	50.0	17	50.0	0.0930
Middle	81	54	66.7	27	33.3	
Lower	70	50	71.4	20	28.6	
Histology						
Well	69	43	62.3	26	37.7	0.7280
Moderate	92	61	66.3	31	33.7	
poor	24	17	70.8	7	29.2	
pT						
T1,2	68	50	73.5	18	26.5	0.0770
T3,4	117	71	60.7	46	39.3	
**pN**						
**N0**	73	58	79.50	15	20.50	**0.0010**
**N1**	112	63	56.30	49	43.70	
**pM**						
**M0**	139	101	72.7	38	27.30	**0.0003**
**M1**	46	20	43.5	26	56.50	
**p-stage**						
**I,II**	88	73	82.9	15	17.10	**0.0000**
**III,IV**	97	48	49.5	49	50.50	
Lymphatic invasion						
negative	58	43	74.1	15	25.9	0.0920
positive	127	78	61.4	49	38.6	
Vascular invasion						
negative	100	69	69	31	31	0.2650
positive	85	52	61.2	33	38.8	

Analysis of 185 therapy naïve ESCC patients (case #1-#185) in **Supplementary Data 1**.
