# Supplementary material for: Interplay between Notch1 and Notch3 promotes EMT and tumor initiation in squamous cell carcinoma
Source: Nat Commun. 2017 Nov 24;8:1758. doi: 10.1038/s41467-017-01500-9 (PMC5700926; doi:10.1038/s41467-017-01500-9)
Supplement: Supplementary file 3 — Description of Additional Supplementary Files [file 41467_2017_1500_MOESM3_ESM.pdf]

Description of Additional Supplementary Files

File Name: Supplementary Data 1
Description: Clinocopathologic ESCC patient data and associated molecular features. Tissues procured from indicated ESCC patients were evaluated for expression of the activated form of NOTCH1 (ICN1) and ZEB1 through immunohistochemistry. NOTCH1 DNA sequencing was performed in indicated subset of patients. See "Patient data key" for information on abbreviations and information on all numerical values.
