# Supplementary material for: Interplay between Notch1 and Notch3 promotes EMT and tumor initiation in squamous cell carcinoma
Source: Nat Commun. 2017 Nov 24;8:1758. doi: 10.1038/s41467-017-01500-9 (PMC5700926; doi:10.1038/s41467-017-01500-9)
Supplement: Supplementary file 2 — Peer Review File [file 41467_2017_1500_MOESM2_ESM.pdf]

Reviewers' comments:

Reviewer #1 (Remarks to the Author):

Brief Summary:
The authors present interesting data about the understanding of how tumor initiation and heterogeneity occurs in squamous cell carcinoma.The authors report a mechanism where Notch1 and Notch 3 may have an opposing function to regulate these processes both in vitro and in vivo. They purport that Notch1 activation and EMT are coupled to promote tumor initiation and cancer cell heterogeneity while the repression of Notch3 by transcription factor ZEB1 limits ICN1-induced differentiation permitting ICN1-mediated EMT. Although the manuscript is scientifically sound and the data is very nice, it appears these mechanisms have already been reported separately, and hence there may be a significant overlap between these data and recently published papers. Which might impact its relevance for NComm verses another venue. The manuscript is well written, it has important clinical message since disease progression via TGFβ-mediated EMT could offer the TGFβ-ZEB1-Notch1 axis as a potential therapeutic target in SCC and should be of great interest to the field and beyond.
Overlap concern derived from the following manuscripts:
- Ohashi, Shinya, et al. "A NOTCH3-mediated squamous cell differentiation program limits expansion of EMT-competent cells that express the ZEB transcription factors." Cancer research 71.21 (2011): 6836-6847.
- Li, Yumei, et al. "Regulation of EMT by Notch signaling pathway in tumor progression." Current cancer drug targets 13.9 (2013): 957-962.
- Natsuizaka, Mitsuteru, et al. "Notch regulates squamous differentiation, cell plasticity and tumor heterogeneity in esophageal carcinoma." Cancer Research 71.8 Supplement (2011): 5194-5194.
- Wang, Tao, et al. "Notch-1-mediated esophageal carcinoma EC-9706 cell invasion and metastasis by inducing epithelial–mesenchymal transition through Snail." Tumor Biology 35.2 (2014): 1193-1201.

Major points to address:
1. If EpCAM is overexpressed in the majority of human epithelial cancers including SCC and plays an important role in EMT. Why do you select tdTomatopos, EpCAMneg instead of EpCAMpos to evaluate the mesenchymal characteristics? If EpCAM participate in TGF-β1-induced EMT.
2. In the figure 3, the authors explain that tdTomatopos, EpCAMneg cells have mesenchymal characteristics, and then they explain (for the same figure) that complete loss of epithelial characteristics was uncommon in 4NQO-induced SCC. It is not clear which cells have mesenchymal characteristics.
3. In the figure 1g the authors explain that when p53 was deleted prior to 4NQO administration, p53 loss did not prevent ICN1 expression in 4NQO-induced lesions; it could be important highlight, differences in the expression of ICN1 between cells (p53+/+ and p53-/-) with 4NQO induction and without 4NQO.
4. In the figure 2e, it could be interesting to corroborate the mesenchymal characteristics in EN60 and TE11 cell lines with high expression of CD44 (H and L) to make a more accurate comparison.
5. In the figure 2e the authors explain that CD44H cells do not require Notch for tumor initiation, but it could add that the tumor initiation is independent of Notch because there are not differences of tumor formation with DOX or without DOX.
6. Why the authors select just TE11 cell line to evaluate if TGFβ influences Notch1-mediated tumor promotion? One cell line is not sufficient to generalize claim.
7. It could be interesting to evaluate the levels of Notch expression using anti-TGFβ blocking antibody.
8. Extension of results to clinically relevant samples such as PDX models would certainly increase enthusiasm.

Overall this is a very nice study and if the authors can address the major reservations of the manuscript the reviewer thinks it would be acceptable for publication.

Reviewer #2 (Remarks to the Author):

The immunostaining data with the chemically induced oral cancer does not does not add much new, and most of the new results are derived subsequently from a variety of cancer cell lines. Using these lines, the authors nicely demonstrate importance of the notch intracellular domain. Although they show ZEB1 repression of Notch3, the linkage between TGF-beta, ZEB1, Notch3 and notch1 is not as clearly demonstrated as shown in the final model. Given the previous extensive study of the molecules examined here, it would have been nice to examine roles for at least some of the molecules in vivo in the chemically induced model, as opposed to cell lines.

The following is a point-by-point response to the helpful and insightful comments of the Reviewers:

Reviewer #1:

“The authors present interesting data about the understanding of how tumor initiation and heterogeneity occurs in squamous cell carcinoma”…“The authors report a mechanism…both in vitro and in vivo...”…“the manuscript is scientifically sound and the data is very nice…“it has important clinical message…and should be of great interest to the field and beyond”…”Overall this is a very nice study and if the authors can address the major reservations of the manuscript the reviewer thinks it would be acceptable for publication…there may be a significant overlap between these data and recently published papers….Overlap concern derived from the following:

- Natsuizaka et al…Cancer Research 71.8 Supplement (2011): 5194-5194.
- Ohashi et al…Cancer Research 71.21 (2011): 6836-6847.
- Wang et al…Tumor Biology 35.2 (2014): 1193-1201.
- Li et al…Current Cancer Drug Targets 13.9 (2013): 957-962…”

We appreciate the positive comments from this Reviewer. With regard to the Reviewer's concern involving potential overlap with published findings, the current manuscript represents a logical extension of our earlier work (Natsuizaka et al., a meeting abstract for the AACR annual meeting and Ohashi et al., an original article in *Cancer Research*) which demonstrated that (1) expression of transcription factor ZEB1 is associated with EMT in esophageal squamous cell carcinoma (ESCC); and (2) Notch3 inhibition may promote EMT. The current manuscript provides new functional and mechanistic insights into the role of Notch1 in carcinogenesis. Specifically, this manuscript (1) defines a novel mechanism through which ZEB1 represses *Notch3* directly to suppress squamous cell differentiation while Notch1 and TGFβ cooperate to promote EMT (de-differentiation); and (2) elucidates how the activated form of Notch1 induces CD44H cells with enhanced malignant potential; and (3) utilizes the single cell-derived 3D neoplastic organoid system as well as cell-lineage tracing experiments to document EMT, for the first time, in a mouse model of ESCC. Additionally, while Wang et al. used a single ESCC cell line to demonstrate that Notch1 influences expression of Snail and other EMT markers in the context of cell migration/invasion *in vitro*, this study failed to establish a mechanistic link between Notch1 and EMT, a knowledge gap that is addressed by the current manuscript. Finally, the mini-review article by Li et al. does not cite any previous studies diminishing the novelty of our current manuscript. We have updated the introduction and discussion sections of the revised manuscript to better describe the studies noted by this Reviewer as they relate to the current manuscript.

Major points to address:

1. "If EpCAM is overexpressed in the majority of human epithelial cancers including SCC and plays an important role in EMT. Why do you select tdTomato-pos, EpCAM-neg instead of EpCAM-pos to evaluate the mesenchymal characteristics? If EpCAM participate in TGF-β1-induced EMT."

Although epithelial cancer cells express EpCAM, they may lose EpCAM expression as a result of EMT mediated by TGFβ present in the tumor microenvironment. Since tumor tissues contain non-epithelial cells (e.g. fibroblasts), EpCAM-negative cells are not necessarily cancer cells. In cell-lineage tracing experiments, epithelial cells are labeled with tdTomato prior to chemically-induced carcinogenesis. By looking at tdTomato-positive tumor cells, we can document EMT in cancer cells even if they no longer express EpCAM. We have clarified this experimental design in the revised text (**Page 5, lines 8-22**).

2. "…the authors explain that tdTomato-pos, EpCAM-neg cells have mesenchymal characteristics, and then they explain (for the same figure) that complete loss of epithelial characteristics was uncommon in 4NQO-induced SCC. It is not clear which cells have mesenchymal characteristics."

The reviewer refers to **Figure 1c** about the data from 4NQO-induced SCC. We defined SCC cell mesenchymal characteristics when two criteria were met: (1) cells are negative for cell surface EpCAM expression and (2) cells exhibit upregulation of mesenchymal markers including *Zeb1* and *Cdh2* (**Figure 1c**). Quantitative RT-PCR for EMT markers was done on EpCAM-negative and EpCAM-positive cells purified by fluorescence-activated cell sorting (FACS) from dissociated tumor cells expressing tdTomato (**Supplementary Figure 1c**). tdTomato expression assures epithelial-cell origin of SCC cells as answered for above question #1 by this reviewer. We postulated that complete loss of epithelial characteristics was uncommon in 4NQO-induced SCC because intratumoral SCC cells display heterogeneity as corroborated by continuous distribution of EpCAM expression (**Supplementary Figure 1c**). Mesenchymal cell markers are detectable in cells with low EpCAM expression. Such findings may be consistent with the epithelial-mesenchymal hybrid phenotype in cancer cells that has been postulated by others [*Sci Signal.* 2014 Sep 30;7(345):ra91. PMID: 25270257 and *Proc Natl Acad Sci U S A.* 2013 Nov 5;110(45):18144-9. PMID: 24154725]. In the revised manuscript, we now discuss such a possible interpretation of our data (**Page 14, lines 24-Page 15, line 5**).

3. "In the figure 1g the authors explain that when p53 was deleted prior to 4NQO administration, p53 loss did not prevent ICN1 expression in 4NQOinduced lesions; it could be important highlight, differences in the expression of ICN1 between cells (p53+/+ and p53-/-) with 4NQO induction and without 4NQO."

We agree. In the original submission, experienced pathologists (AJK and SN) scored independently ICN1 expression in 4NQO-induced tumors from mice with or without targeted p53-deletion in the esophagus. The ICN1 labeling index (**Figure 1g**) was determined based on the frequency of ICN1-positive SCC cells within each tumor examined. We examined also ICN1 expression in mice with or without esophagus targeted p53-deletion in the absence of 4NQO treatment; however, there was no difference noted in ICN1 expression level between $p53^{+/+}$ and $p53^{-/-}$ cells and that ANOVA with Tukey's post-hoc test failed to detect a significant difference ($p<0.05$) in the ICN1 labeling index between groups (three animals per group) when $p53^{+/+}$ and $p53^{-/-}$ cells were compared in normal esophageal mucosa without 4NQO treatment. As these data represent a negative result, we did not include in the original manuscript. For the revised manuscript, we now report these findings (**Page 6, lines 16-20**) along with a reference [*Mol Cell Biol.* 2007 May;27(10):3732-42. PMID: 17353266] reporting a consistent finding that p53 deficiency alone does not impair basal Notch1 expression as well as Notch1-mediated squamous-cell differentiation in mice.

4. "…it could be interesting to corroborate the mesenchymal characteristics in EN60 and TE11 cell lines with high expression of CD44 (H and L) to make a more accurate comparison".

In revised **Figure 3a** and **Supplementary Figure 2a and 2b**, we demonstrate that CD44H cells express mesenchymal characteristics compared to CD44L cells within the tumors for both cell lines. We now describe these observations in the revised text (**Page 7, line 24-Page 8, Line 2**).

5. "…the authors explain that CD44H cells do not require Notch for tumor initiation, but it could add that the tumor initiation is independent of Notch because there are not differences of tumor formation with DOX or without DOX".

We agree that tumor initiation by CD44H cells may be independent of Notch because purified CD44H cells gave rise to tumors with or without DOX-induced ICN1 expression or in the presence of DNMAML1, a genetic pan-Notch inhibitor (revised **Figure 3c**). We now state this explicitly in the revised Results (**Page 8, line 15**) and discussed (**Page 15, lines 11-15**).

6. "Why the authors select just TE11 cell line to evaluate if TGFβ influences Notch1-mediated tumor promotion? One cell line is not sufficient to generalize claim".

In the revised manuscript, we now demonstrate that TGF-β inhibition via the TGFβ neutralizing therapeutic antibody 1D11 suppresses growth of patient-derived xenograft (PDX) tumors from head and neck squamous cell carcinoma (HNSCC)(**Figure 4a**), indicating that this finding is not specific to TE11 ESCC cells and may be more generalizable to SCCs beyond ESCC.

7. "It could be interesting to evaluate the levels of Notch expression using anti-TGFβ blocking antibody".

We agree. In the revised manuscript, we have evaluated the effects of the TGFβ neutralizing antibody 1D11 in 4NQO-induced murine esophageal lesions (**Figure 4b**) where administration of 1D11 diminished significantly the ICN1 level. Additionally, 1D11 decreased the frequency of ESCC and precursor lesions and EMT (**Figure 4c**).

8. Extension of results to clinically relevant samples such as PDX models would certainly increase enthusiasm.

As answered for the concern expressed by this reviewer in the above point #6, we have analyzed the effect of the TGFβ neutralizing therapeutic antibody 1D11 on a HNSCC PDX and report impairment of tumor growth (**Figure 4a**) in the revised manuscript.

Reviewer 2:

"*The immunostaining data with the chemically induced oral cancer does not add much new, and most of the new results are derived subsequently from a variety of cancer cell lines.*"

Our findings using immunostaining in 4NQO-induced ESCC coupled with cell-lineage tracing experiments are the first unequivocal documentation of EMT in squamous-cell carcinomas. Additionally, ICN1 IHC data in mice and human SCC patients provides insights into Notch1 activation status as it relates to the natural history of SCC development and progression. *Notch1* deletion in the murine skin results in non-cell autonomous SCC development due to inflammation associated with epidermal barrier defects [Cancer Cell. 2009 Jul 7;16(1):55-66. PMID: 19573812]. Such an approach limits the assessment of the cell-autonomous oncogenic role of Notch1 *in vivo*. We have utilized the three-dimensional (3D) organoid system where a tissue-derived single cell suspension gives rise to spherical structures with recapitulation of the histopathology present in the original *in situ* neoplastic lesions. Generating 3D organoids from $Notch1^{loxP/loxP}$ mice with 4NQO-induced ESCC and precursor lesions, we now demonstrate functional consequences of Notch1 deletion *ex vivo* to find that Notch1 plays a role in the initiation of neoplastic organoid formation (**Figure 2f**) and EMT (**Figure 3e**). We now highlight the relevance of these findings to SCC biology in the revised discussion.

"Using these lines, the authors nicely demonstrate importance of the notch intracellular domain. Although they show ZEB1 repression of Notch3, the linkage between TGFβ, ZEB1, Notch3 and notch1 is not as clearly demonstrated as shown in the final model. Given the previous extensive study of the molecules examined here, it would have been nice to examine roles for at least some of the molecules in vivo in the chemically induced model, as opposed to cell lines".

We acknowledge the Reviewer's concern regarding evaluation of the roles of TGFβ, Notch1, Notch3 and Zeb1 *in vivo*. In the revised manuscript, we now evaluate the influence of the TGFβ neutralizing therapeutic antibody 1D11 upon Notch signaling and EMT in the context of 4NQO-mediated carcinogenesis (**Figure 4b, c; Supplementary Figure 3e**), providing evidence that TGFβ supports ICN1 expression and EMT *in vivo*. To examine further the functional role of Notch1 in Zeb1 expression and EMT, we have utilized 4NQO-induced neoplastic 3D organoids from animals expressing $Notch1^{loxP/loxP}$ for *ex vivo* recombination experiments (**Figure 2f and Figure 3e**) as described above.

In summary, we believe that the manuscript is much improved and are appreciative of the helpful comments of the Reviewers. We hope you find the revised manuscript suitable for publication.

REVIEWERS' COMMENTS:

Reviewer #1 (Remarks to the Author):

No further comments. Authors addressed reviews

Reviewer #2 (Remarks to the Author):

A major concern with the original paper was the lack of in vivo evidence for a role for Notch1/3. The authors have responded with an assessment of anti-Tgfb antibody in the chemical model. It is unclear whether the sole role of Tgfb is aimed at Notch1/3, so this analysis is less informative than one aimed instead at the Notch pathway per se. Nevertheless, the results do show an important role for Tgfb in tumor progression. And coupled with their extensive analysis of the pathway in cell culture, the revised manuscript is now improved.

Response to Reviewer 2's remaining concern:

Reviewer 2: "*A major concern with the original paper was the lack of in vivo evidence for a role for Notch1/3. The authors have responded with an assessment of anti-Tgfb antibody in the chemical model. It is unclear whether the sole role of Tgfb is aimed at Notch1/3, so this analysis is less informative than one aimed instead at the Notch pathway per se. Nevertheless, the results do show an important role for Tgfb in tumor progression. And coupled with their extensive analysis of the pathway in cell culture, the revised manuscript is now improved*".

We are grateful for this Reviewer's positive note. While our data from our cell culture studies support Notch1 as a downstream effector of transforming growth factor-β (Tgfb), we agree that the observed inhibitory effects of anti-Tgfb antibody upon tumor progression in mice may involve Tgfb downstream effectors other than Notch. This poses a limitation of our experimental design in mice and is now discussed in the revised manuscript (page 16, lines 6-8). The complexity of the role of Notch signaling in tumor biology is echoed by that of Tgfb as intratumoral cancer cell heterogeneity represents cancer cells that can respond to Tgfb and those that cannot, the latter emerging during disease progression (PMID: 24132110)(page 14, lines 21-23). Although we have demonstrated that murine squamous cell carcinoma (SCC) and precancerous lesions express the activated form of Notch1 (**Figure 1 and Supplementary Figure 1**), it is currently difficult to evaluate the oncogenic properties of Notch1 by simply deleting *Notch1* in the murine squamous epithelia *in vivo*. Notch inhibition may permit clonal immortalization and expansion of murine esophageal epithelial cells (PMID: 24814514)(page 15, line 26-page 16 line 1). Additionally, Notch inhibition may perturb squamous-cell differentiation and cause epithelial barrier defects, facilitating the tumor-promoting inflammatory microenvironment (PMID: 19573812). Therefore, *Notch1* deletion may reveal a tumor suppressor function for Notch. To evaluate the functional consequences of *Notch1* deletion in the absence of non-epithelial (stromal) cell components, we analyzed novel *ex vivo* 3D organoids generated from 4NQO-induced esophageal epithelial lesions. We demonstrate that *Notch1* deletion prevents neoplastic 3D organoid formation as well as EMT (**Figures 2f and 3e**). The 4NQO model requires long-term 4NQO administration (16 weeks) and cancer or precancerous esophageal lesions are not detected morphologically immediately following 4NQO treatment. Tumors arise 6-9 weeks after 4NQO withdrawal; however, it is unclear precisely when malignant transformation occurs in this model. Single cell-derived 3D organoids have the potential to detect neoplastic changes in a more sensitive and quantitative manner than conventional morphological tissue assessment. Such a study is underway, albeit beyond the scope of this manuscript. Once we determine an appropriate window of 4NQO-induced malignant transformation, we will use cell-lineage traceable mice (i.e. $K5Cre^{ERT2}$;$R26tdTomato^{lsl/lsl}$; $Notch1^{loxP/loxP}$ mice) following 4NQO administration to elucidate how *Notch1* may exert its oncogenic role by promoting EMT and tumor initiation and/or progression *in vivo*. Alternatively, such mice may be treated with Notch1 specific antagonistic antibody (PMID: 20393564) or anti-Notch3 agonistic antibody (PMID: 18182388), the latter can be used for targeted activation of Notch3. Such a future perspective is now added into the revised discussion (page 17 lines 2-14). We hope that these additions to the manuscript address the remaining concern of Reviewer 2.
